# Versican regulating viscoelasticity drives pleural fibrosis via mechanotransductive signaling

**DOI:** 10.1172/jci.insight.199507

**Published:** 2026-04-23

**Authors:** Zi-Heng Jia, Xin-Liang He, Xiao-Lin Cui, Qian Li, Pei-Pei Cheng, Li-Qin Zhao, Shu-Yi Ye, Shi-He Hu, Chen-Yue Lian, He-De Zhang, Li-Mei Liang, Lin-Jie Song, Fan Yu, Liang Xiong, Fei Xiang, Xiaorong Wang, Meng Wang, Xiyong Dai, Hong Ye, Wan-Li Ma

**Affiliations:** 1 Department of Respiratory and Critical Care Medicine, Union Hospital, Tongji Medical College, Huazhong University of Science and Technology, Wuhan, China.; 2 Key Laboratory of Respiratory Diseases of National Health Commission of China, Wuhan, China.; 3 Department of Pathophysiology, School of Basic Medicine, Tongji Medical College, Huazhong University of Science and Technology, Wuhan, China.; 4 Department of Thoracic Surgery, Wuhan Pulmonary Hospital, Wuhan, China.

**Keywords:** Cell biology, Inflammation, Fibrosis

## Abstract

Extracellular matrix (ECM) disorder was believed to result from fibrosis, but it has recently been recognized that fibrotic ECM initiates a self-reinforcing circuit and contributes to the development of fibrosis. Versican, an ECM component, participates in cell-ECM interaction and ECM regeneration. In pleura, versican is primarily derived from pleural mesothelial cells (PMCs). However, the role and mechanism of versican in pleural fibrosis has remained unknown. In this study, versican and versican-mediated pleural viscoelasticity were found to be elevated in both human and murine pleural fibrotic tissues. Versican knockdown by shRNA prevented increases in viscoelasticity as well as pleural fibrosis. High levels of versican and viscoelasticity promoted mesothelial-mesenchymal transition in PMCs. Mechanistically, increased viscoelasticity induced pleural fibrosis through the CD44/USP10/Smad4 mechanotransduction pathway. In conclusion, these results revealed that excessive versican in fibrotic pleural ECM enhanced ECM viscoelasticity and consequently promoted progression of pleural fibrosis.

## Abstract 2

This study revealed that excessive versican in fibrotic pleural ECM enhanced ECM viscoelasticity, and consequently promoted progression of pleural fibrosis.

## Graphical abstract

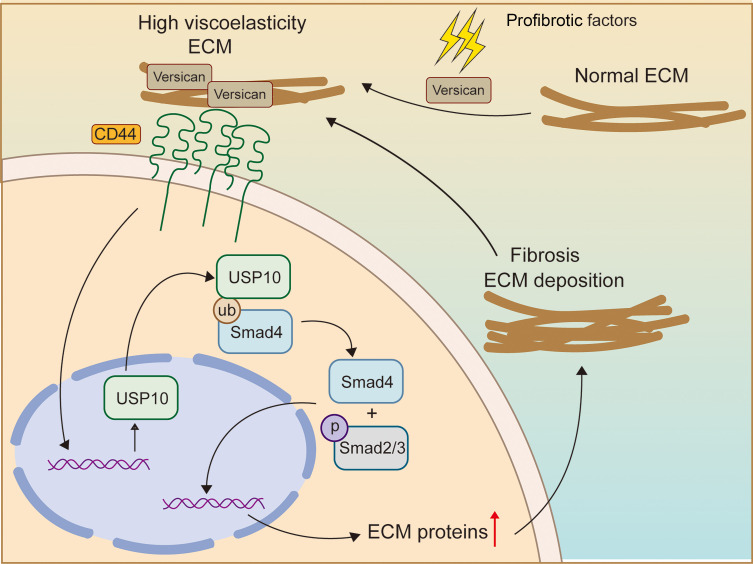

## Introduction

The pleura is a membrane with a smooth and lubricating surface. It forms the pleural cavity that covers the pulmonary outer surface and the inner wall of the thoracic cavity. The pleura plays a role in protecting and supporting the lung (1). The majority of the pleura is composed of pleural mesothelial cells (PMCs). At the same time, PMCs are involved in the formation of the physiological architecture and function of the pleura. PMCs also play a central role in pathological processes of the pleura.

Pleural fibrosis is a disorder with excessive accumulation of extracellular matrix (ECM), leading to the disruption of pleural architecture and impairment of lung function. The primary causes of pleural fibrosis include tuberculous pleurisy, asbestos-induced injury, and rheumatoid pleurisy (2). The manifestation of pleural fibrosis includes discrete localized lesions known as pleural plaques and extensive pleural thickening accompanied by fibrosis (3). The crosstalk between pleura and various mediators, including immune effector cells, cytokines, and blood-derived proteins, is related to the pathogenesis of pleural fibrosis (3). A study of pleural fibrosis treatment focused on growth factors and signal pathways (4). However, pleural fibrosis has been considered irreversible, with the only solution being surgical dissection.

Disorders of ECM were previously believed to result from fibrotic diseases. However, ECM has recently been recognized as a key mediator in fibrosis, including cardiac fibrosis, liver fibrosis, and pulmonary fibrosis (5–9). It is currently believed that after the initiation of fibrosis, the abnormal ECM microenvironment and abnormal continuous activation of myofibroblasts constitute a positive feedback loop that promotes fibrosis, leading to the self-maintenance and continuous progression of fibrosis (10, 11). The progression of fibrosis cannot be restrained by simply targeting initiators of fibrosis without amending ECM.

The components of fibrotic ECM diverge from those of normal tissue ECM. Versican, as a constituent of ECM, serves a crucial role in cell-ECM crosstalk (12, 13), especially during ECM remodeling (14, 15). Aortic accumulation of the proteoglycan versican has been found as the main contributor to thoracic aortic aneurysm and dissection in Marfan syndrome (16). Glycosaminoglycan chains, which are negatively charged molecules, are covalently linked to the protein core of versican. These chains give the aorta its flexibility, compressive resilience, and viscoelasticity (17). However, the exact role of versican in pleural fibrosis needs to be clarified.

Aberrant deposition of ECM in fibrosis changes the tissue mechanical force, increasing stiffness and viscoelasticity and decreasing shear force and elasticity (18, 19). Conversely, mechanical stresses can promote abnormal tissue restructuring and fibrosis when repair mechanisms fail or injury recurs. Increased stiffness has been reported to induce development of fibrosis (19). As a large proteoglycan, versican has been found to increase the viscoelasticity of ECM (20, 21). Furthermore, changes in ECM viscoelasticity affect cellular behaviors and the disease process, for instance, in breast cancer and hepatocellular carcinoma (22–24). So far, the role of increased ECM viscoelasticity in the progression of fibrosis has remained unknown.

Our study was designed to explore how abnormal ECM contributes to the progression of pleural fibrosis caused by tuberculous pleurisy. By using tandem mass tag proteomic profiling, we identified versican as a potential player in the progression of pleural fibrosis and investigated the underlying mechanisms.

## Results

### Tuberculous pleural ECM-mediated profibrogenic phenotype of PMCs.

**Figure 1:**
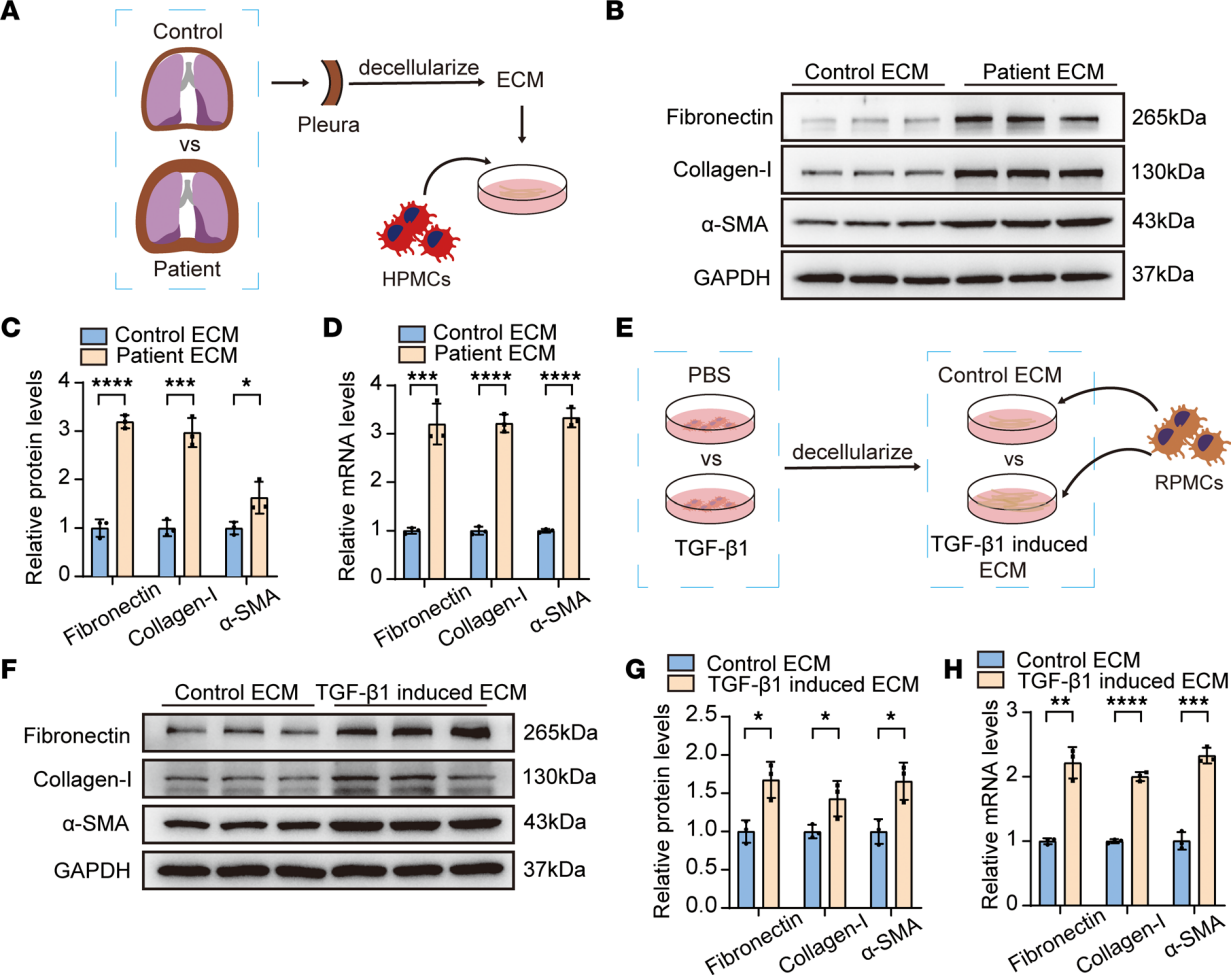
Fibrotic pleural ECM mediated profibrogenic phenotypes of PMCs. (**A**) Diagram shows the preparation of acellular pleural ECM from patients with tuberculous pleural fibrosis and control group. (**B**–**D**) Primary human PMCs were isolated and cultured with the acellular pleural ECM. After 24 hours, the cells were harvested. Relative protein and mRNA levels of fibronectin, collagen I, and α-SMA were detected by Western blotting (**B** and **C**) and quantitative real-time PCR (qRT-PCR) (**D**). Control group, *n* = 3; patients, *n* = 3. (**E**) Primary rat PMCs were isolated and cultured without or with TGF-β1 for 24 hours. Then, rat PMC ECM was prepared by decellularization. (**F**–**H**) New primary rat PMCs were treated with TGF-β1–induced ECM or control ECM for 24 hours. Relative protein and mRNA levels of fibronectin, collagen I, and α-SMA were detected by Western blotting (**F** and **G**) and qRT-PCR (**H**). Data are presented as mean ± SEM. Statistical analysis was performed by unpaired Student’s *t* tests. *n* = 3, *P < 0.05, **P < 0.01, ***P < 0.001, ****P < 0.0001.

To explore whether tuberculous pleural ECM contributes to pleural fibrosis, acellular pleural ECM was prepared by decellularizing tuberculous pleura and normal control pleura. Primary human PMCs were identified (Supplemental Figure 1; supplemental material available online with this article; https://doi.org/10.1172/jci.insight.199507DS1) and seeded into the acellular pleural ECM (Figure 1A). As shown in Figure 1, B and C, and Supplemental Figure 2, the acellular pleural ECM from tuberculous pleura increased protein expression of fibronectin, collagen I, and α–smooth muscle actin (α-SMA) in PMCs, which indicated PMCs were undergoing mesothelial-mesenchymal transition (MesoMT). Consistently, qRT-PCR analysis demonstrated increasing mRNA levels of fibronectin, collagen I, and α-SMA in the cells exposed to the acellular pleural ECM from tuberculous pleura (Figure 1D). To confirm the above results, primary rat PMCs were isolated and cultured without or with TGF-β1 for 24 hours. Then, rat PMC ECM was prepared by decellularization, and TGF-β1–induced ECM and control ECM were obtained and used to culture new primary rat PMCs (Figure 1E). As shown in Figure 1, F–H, TGF-β1–induced ECM treatment increased protein and mRNA levels of fibronectin, collagen I, and α-SMA, indicating MesoMT in PMCs. Collectively, these data indicated that tuberculous pleural ECM and fibrotic ECM promoted PMC MesoMT.

### Versican expression is upregulated in pleural fibrosis.

**Figure 2:**
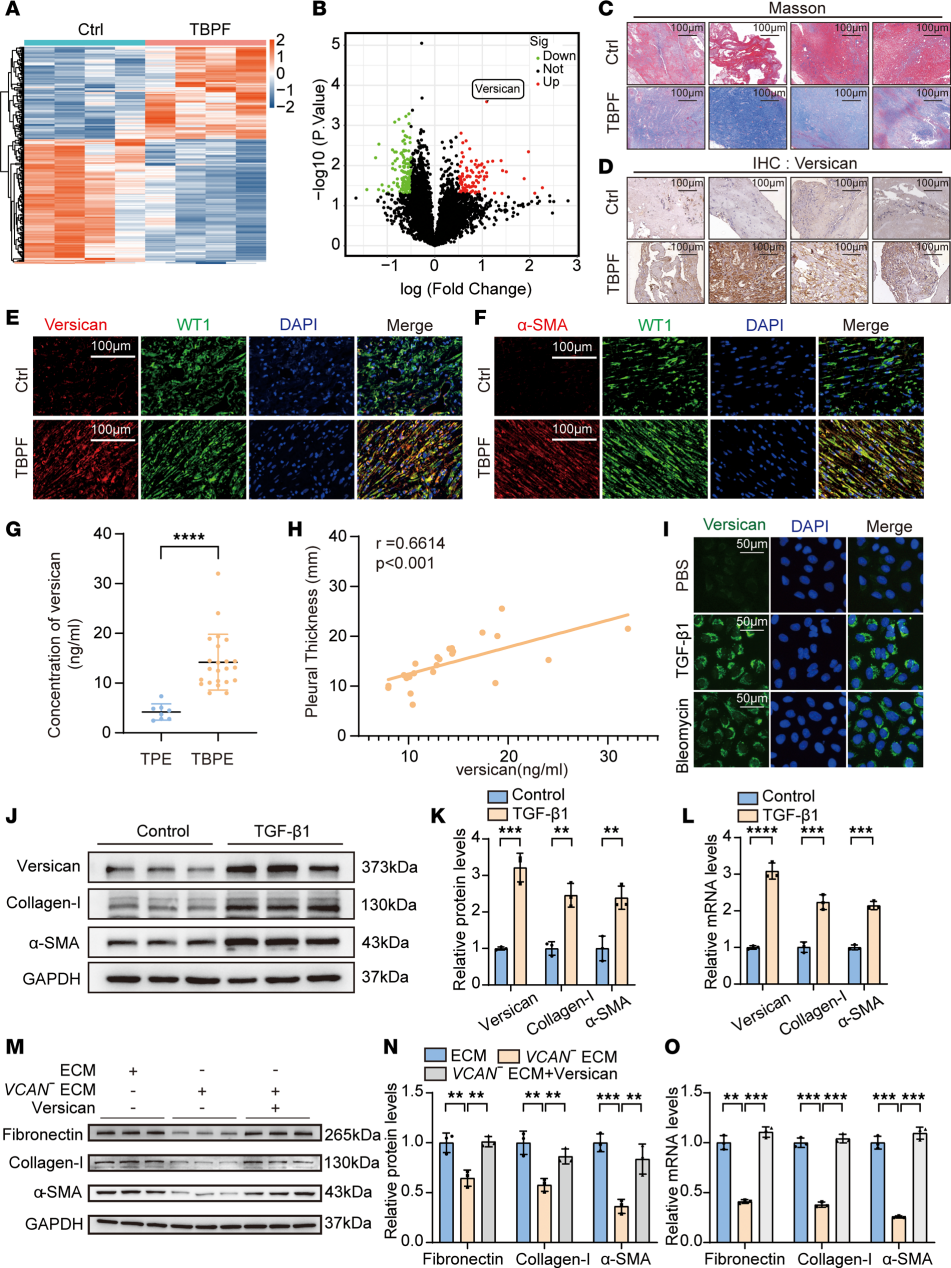
Versican expression was upregulated in pleural fibrosis. (**A**) Protein profile of human fibrotic pleura was performed by tandem mass tag–based proteomics. Ctrl, pleura from control group; TBPF, pleura from patients with tuberculous pleural fibrosis. (*n* = 4). (**B**) Volcano plot of differentially expressed genes. (**C**) Representative images of Masson’s trichrome staining in pleura from control group and patients with TBPF. Scale bars: 100 μm. (**D**) Representative images of IHC staining of versican in pleura of controls or patients. Scale bar: 100 μm. (**E**) Representative images of immunofluorescence staining of versican (red) and Wilms tumor 1 (green). Scale bars: 100 μm. (**F**) Representative images of immunofluorescence images of α-SMA (red) and Wilms tumor 1 (green). Scale bars: 100 μm. (**G**) Pleural effusion versican content by ELISA. (**H**) Correlation analysis between versican concentration in effusion and pleural thickness in patients with TBPF, *r* = 0.6614, *P* < 0.001. (**I**) Representative images of immunofluorescence staining of versican in PMCs incubated with TGF-β (5 ng/mL) or bleomycin (0.2 μg/mL) for 24 hours. (**J**–**L**) PMCs were incubated with TGF-β (5 ng/mL) for 24 hours, after which intracellular protein levels of versican, collagen I, and α-SMA were measured by Western blotting; mRNA levels of versican, collagen I, and α-SMA were detected by qRT-PCR at 12 hours. Data are presented as mean ± SEM. Statistical analyses were performed with unpaired Student’s *t* tests. *n* = 3, **P < 0.01, ***P < 0.001, ****P < 0.0001. (**M**–**O**) Primary rat PMCs were isolated and cultured with *VCAN* siRNA or control siRNA with TGF-β1 for 36 hours. Then, rat PMC ECM was prepared by decellularization. PMCs were treated by control ECM or versican-deficient ECM with or without recombinant versican (1 μg/mL) for 24 hours, after which fibronectin, collagen I, and α-SMA were detected by Western blotting (**M** and **N**) or qRT-PCR (**O**). Statistical analysis was performed by 1-way ANOVA. *n* = 3, **P < 0.01, ***P < 0.001, ****P < 0.0001.

Using tandem mass tag–based proteomic analysis, the protein profile of pleura was investigated based on samples from 4 patients with tuberculous pleural fibrosis (TBPF) and 4 control individuals. Versican, an ECM protein, was increased in TBPF (Figure 2A). For visualization in a volcano plot, the raw *P* values were transformed using –log_10_(*P* value) (Figure 2B). Then, Masson’s trichrome staining was performed to show collagen in pleura samples from patients with TBPF and control participants (Figure 2C). Versican expression was further examined by tissue staining in the pleura samples. As shown in Figure 2, D–F, IHC and immunofluorescence staining showed that versican was almost undetectable in control pleura, whereas it was substantially increased in the pleura from patients with TBPF with high-level expression of α-SMA. Furthermore, versican protein was colocalized with the protein Wilms tumor 1, a marker of PMCs. More samples were examined that confirmed a marked increase of versican expression in the pleura of patients with TBPF (Supplemental Figure 3). The level of versican was higher in tuberculous pleural effusion than in transudative pleural effusion (Figure 2G). Moreover, the concentration of versican in the pleural effusion was positively correlated with pleural thickness (Figure 2H). At the same time, in vitro immunofluorescence, Western blotting, and qRT-PCR analysis showed that TGF-β1 and bleomycin substantially increased the expression of versican with increased protein collagen I and α-SMA in PMCs (Figure 2, I–L, and Supplemental Figure 4, A–C). Then, acellular fibrotic ECM with or without knockdown of versican was prepared by treating PMCs transfected with versican siRNA or control siRNA (Supplemental Figure 4, D–F) with TGF-β. After decellularization, new PMCs were seeded in versican-deficient ECM or control ECM. The control ECM induced upregulation of fibronectin, collagen I, and α-SMA; versican- deficient ECM attenuated these increases (Figure 2, M–O). Moreover, administration of recombinant versican restored the changes (Figure 2, M–O). These data indicated that versican mediated fibronectin, collagen I, and α-SMA synthesis in PMCs in vitro.

The pleura is mainly composed of PMCs and a few fibroblasts. To confirm whether the main source of versican is PMCs, primary human and rat PMCs and fibroblasts were examined. As shown in Supplemental Figure 4, G and H, the basic expression level of versican in primary human PMCs was higher than that in primary human lung fibroblasts. Bleomycin induced versican expression in primary human PMCs, primary human lung fibroblasts, rat PMCs, and rat lung fibroblasts. Moreover, the upregulation of versican in primary human PMCs in response to bleomycin was much greater than that in primary human lung fibroblasts (Supplemental Figure 4G). Consistently, similar results were found in primary rat PMCs and rat lung fibroblasts (Supplemental Figure 4H). These data indicated that versican was upregulated in pleural fibrosis, and it was mainly from activated PMCs.

### Loss of versican reduced viscoelasticity and attenuated pleural fibrosis.

**Figure 3:**
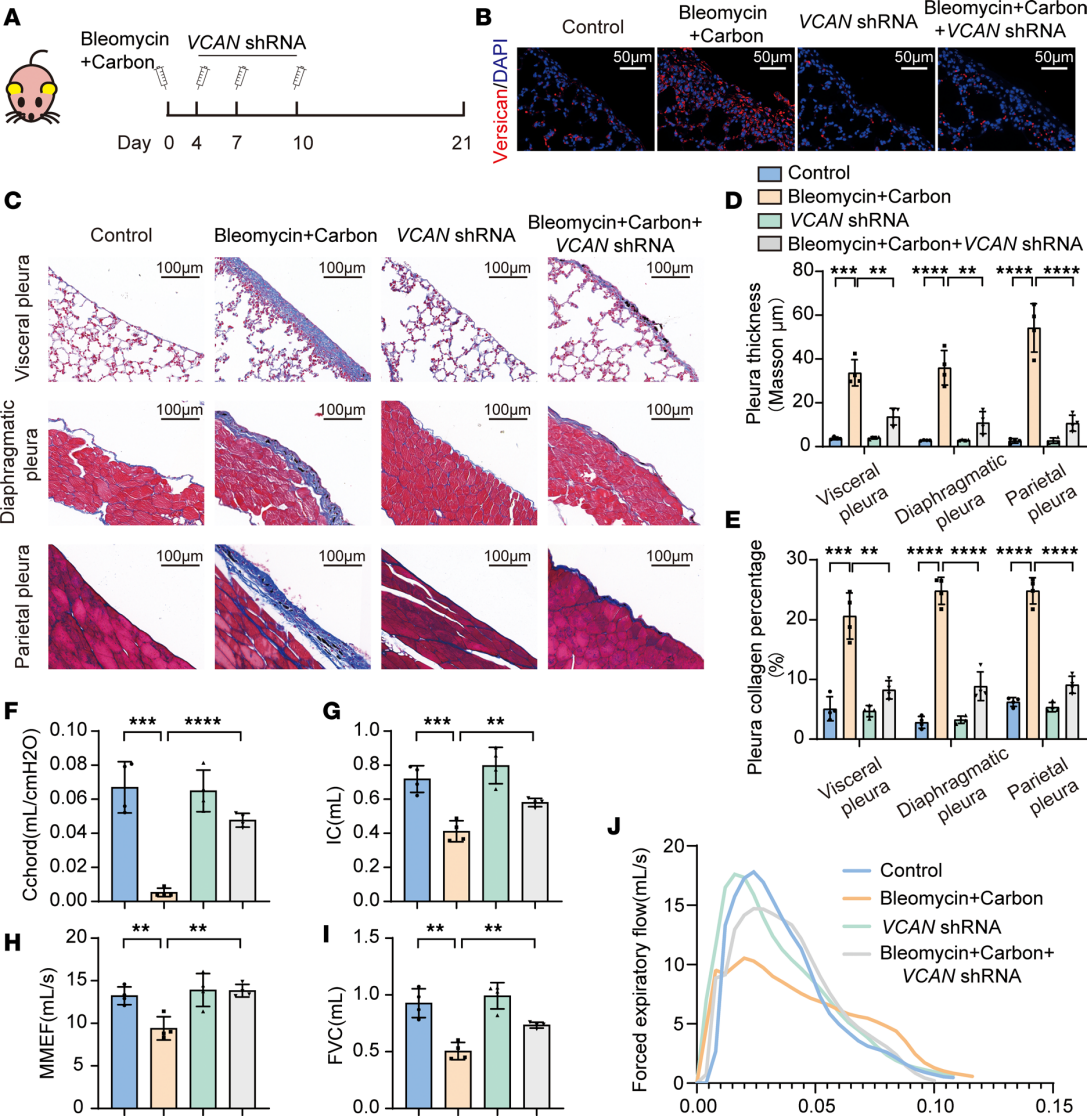
Versican mediated pleural fibrosis in vivo. (**A**) Mouse pleural fibrosis model was induced by intrapleural injection of bleomycin (0.48 μg/mouse) plus carbon particles (0.1 mg/mouse). Lentivirus expressing shRNA directed against versican (VCAN) shRNA or scrambled sequence shRNA was administrated by intrapleural injection at a dose of 2 × 10^6^ TU on days 4, 7, and 10. After a lung function test, all mice were euthanized at day 21, and then tissues were taken for analysis. (**B**) Immunofluorescence staining of versican in visceral pleura of mouse pleural fibrosis model. Scale bars: 50 μm. (**C**) Representative Masson’s trichrome staining images of visceral pleura from lung sections, parietal pleura from chest wall, and diaphragm sections. Scale bar: 100 μm. (**D**) Changes in pleural thickness. (**E**) Changes in collagen percentages of pleura. (**F**–**J**) Changes in lung function. Cchord, chord compliance.

To verify whether versican mediates pleural fibrosis, mouse pleural fibrosis models were made by intrapleural injection of bleomycin plus carbon particles (Figure 3A). At the same time, versican shRNAs loaded into lentivirus were used to knock down versican (Figure 3B and Supplemental Figure 5). Bleomycin plus carbon induced mouse pleural thickening and fibrosis in visceral, parietal, and diaphragmatic pleura, and versican shRNA attenuated pleural fibrosis (Figure 3, C–E). The versican shRNA also restored declines in mouse lung function, including chord compliance, inspiratory capacity, mean mid-expiratory flow, forced vital capacity, and forced expiratory flow (Figure 3, F–J).

To confirm whether versican mediates fibrotic changes in PMCs in vitro, bleomycin and TGF-β1 were used to treat PMCs. We found that bleomycin and TGF-β1 induced upregulation of fibronectin, collagen I, and α-SMA mRNA as well as their proteins, which were attenuated by versican siRNA (Supplemental Figure 6, A–F). At the same time, recombinant versican proteins were used to treat PMCs. As shown in Supplemental Figure 6, G–I, versican increased mRNA and protein levels of fibronectin, collagen I, and α-SMA.

**Figure 4:**
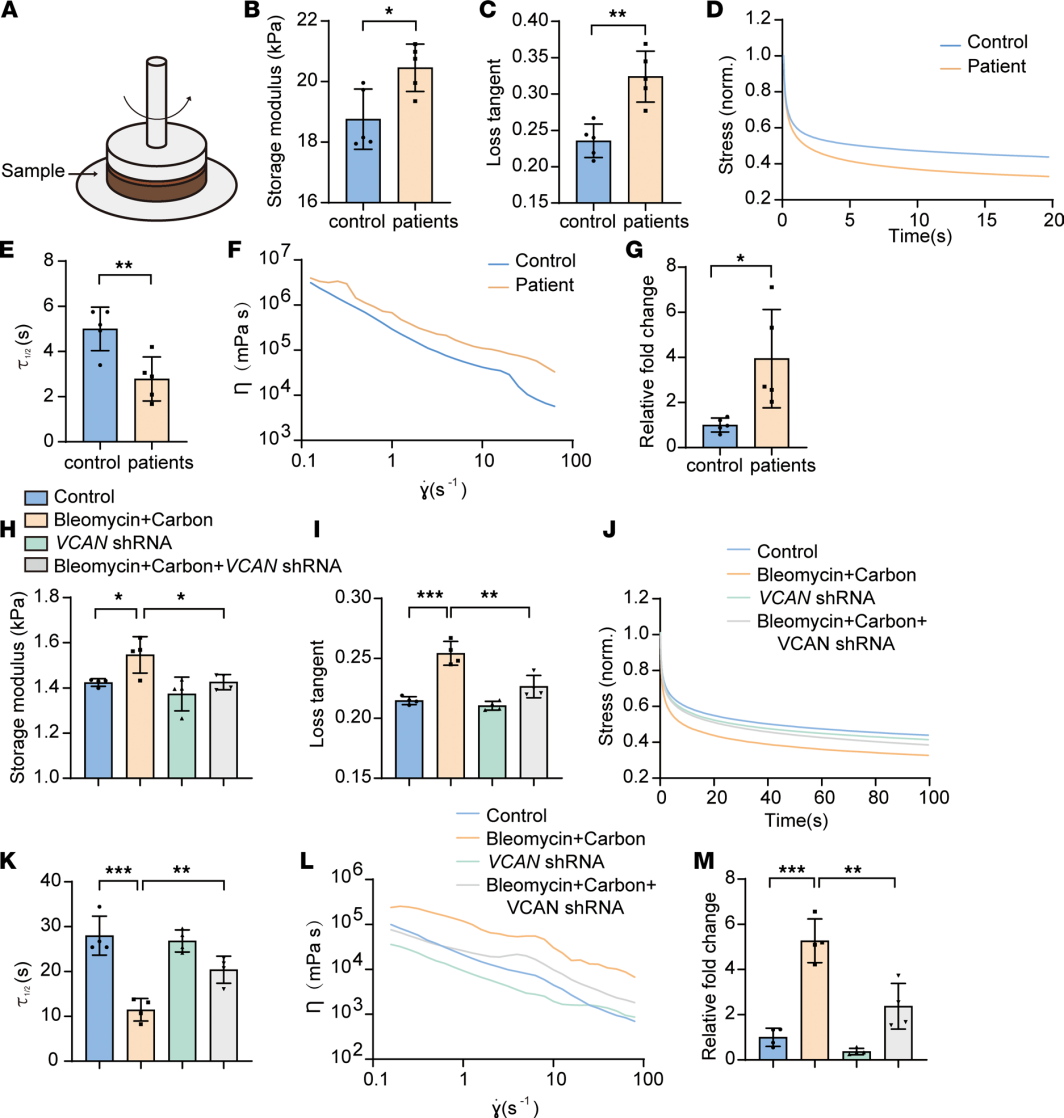
Loss of versican reduced viscoelasticity of fibrotic pleura. (**A**) Schematic of rheometry analysis of fresh pleural tissues. (**B**) Rheometry analysis of the storage modulus in control and fibrotic pleura. (**C**) The loss tangent (viscoelasticity) in control and fibrotic pleura. (**D**) Stress relaxation curves in pleura samples from control and patient groups. Norm., normalized. (**E**) Stress was normalized to the initial stress and depicted as τ1/2 (the timescale at which the stress is relaxed to half its original value). (**F**) Pleural tissues were taken from patients with pleural fibrosis and controls. Shear viscosity was detected and corresponding curves were made. (**G**) Bar graphs showing relative fold-changes in viscosity with shear rate at 10 (s^–1^). (**B**, **C**, **E**, and **G**) Data are presented as mean ± SEM. Statistical analyses were performed with unpaired Student’s *t* tests. *n* = 5, *P < 0.05. (**H**–**M**) Mouse pleural fibrosis model was induced by intrapleural injection of bleomycin plus carbon particles. Lentivirus expressing shRNA directed against versican (VCAN) shRNA or scrambled sequence shRNA was administrated by intrapleural injection at a dose of 2 × 10^6^ TU on days 4, 7, and 10. After a lung function test, all mice were euthanized at day 21, and then tissues were taken for analysis. (**H**) Rheometry analysis of the storage modulus. (**I**) The loss tangent (viscoelasticity) in mouse pleura. (**J**) Stress relaxation curves in mouse pleura. (**K**) Stress was normalized to the initial stress and depicted as τ1/2 (the timescale at which the stress is relaxed to half its original value). (**L**) Shear viscosity was detected and corresponding curves were made. (**M**) Bar graphs showing relative fold-changes in viscosity with shear rate at 10 (s^–1^). (**H**, **I**, **K**, and **M**) Data are presented as mean ± SEM. Statistical analyses were performed with 1-way ANOVA. *n* = 4, **P < 0.01, ***P < 0.001.

The above results suggested that increased versican in fibrotic ECM in turn mediated fibrotic changes in pleura. The underlying mechanisms were further explored. It has been reported that versican increases tissue viscoelasticity (25, 26). Here, rheometry was used to evaluate the viscoelasticity of pleural ECM. As shown in Figure 4, A–G, we confirmed that the pleura of patients with TBPF had a higher loss tangent and faster stress relaxation under a constant deformation. The pleura of patients with pleural fibrosis had higher viscoelasticity than that of control pleura at an identical shear rate. The increased viscoelasticity was also found in visceral pleura of a mouse pleural fibrosis model induced by intrapleural injections of bleomycin plus carbon particles. Knockdown of versican with shRNA lentivirus attenuated the changes in viscoelasticity (Figure 4, H–M). Together, these data indicated that versican in fibrotic ECM increased the viscoelasticity of pleura, which might be related to pleural fibrosis.

### Increased viscoelasticity of pleural ECM promoted pleural fibrosis through CD44.

**Figure 5:**
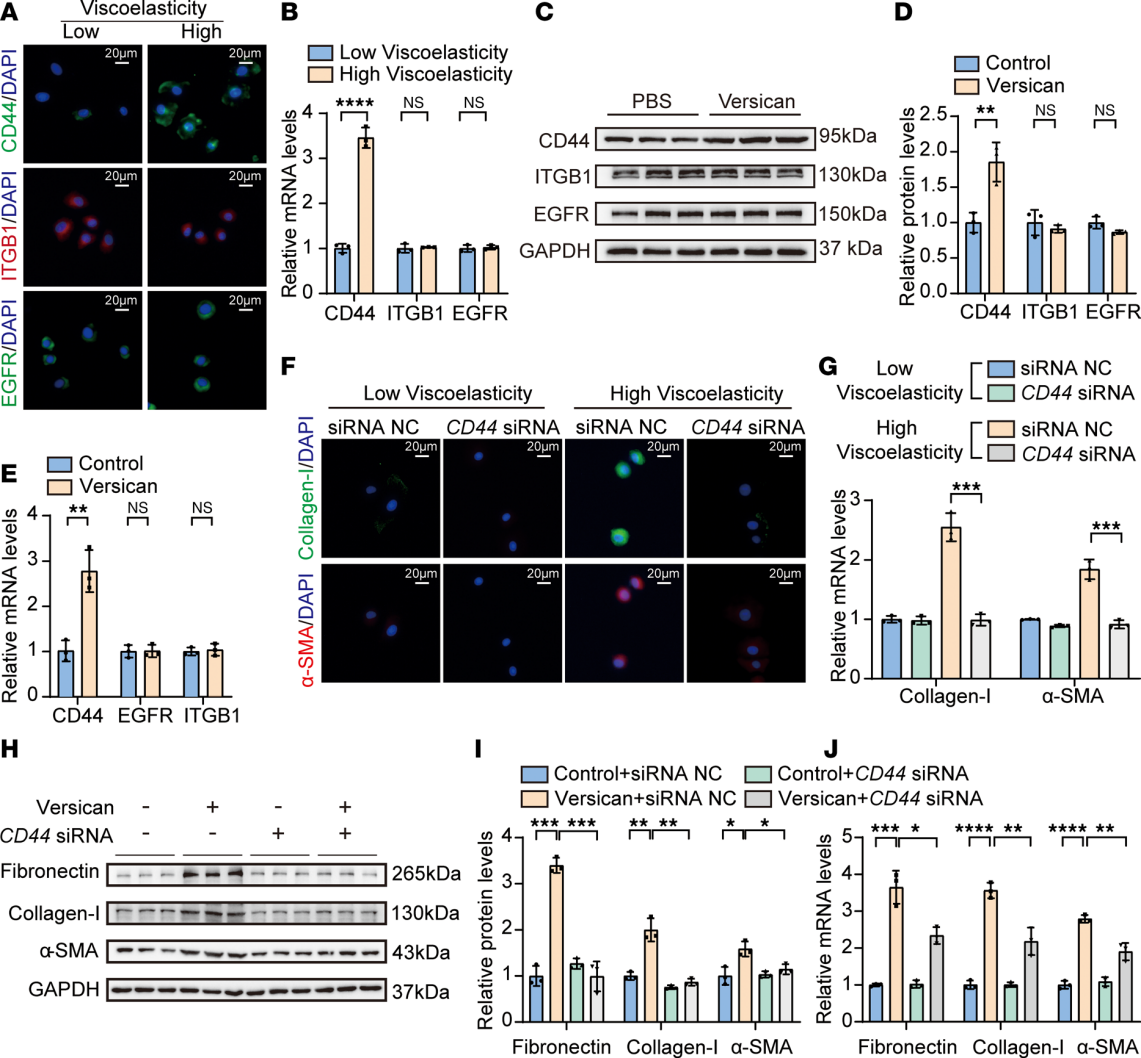
CD44 upregulated and mediated fibrotic phenotype in PMCs. (**A** and **B**) PMCs were cultured in low- or high-viscoelasticity hydrogels for 24 hours. Then PMCs were harvested for immunostaining (**A**) and qRT-PCR (**B**) to detect protein and mRNA expression of CD44, integrin β1 (ITGB1), and EGFR. (**C** and **D**) PMCs were treated by recombinant versican (1 μg/mL) for 24 hours, after which CD44, ITGB1, and EGFR were detected by Western blotting. (**E**) qRT-PCR analysis of CD44, ITGB1, and EGFR expression in PMCs treated with recombinant versican (1 μg/mL) for 12 hours. (**B**, **D**, and **E**) Data are presented as mean ± SEM. Statistical analyses were performed with unpaired Student’s *t* tests. *n* = 3, **P < 0.01, ***P < 0.001. (**F** and **G**) After transfection with *CD44* siRNA (siRNA3 was selected and used) for 36 hours, PMCs were cultured in low- or high-viscoelasticity hydrogels for 48 hours. Then PMCs were harvested for immunostaining (**F**) and qRT-PCR (**G**) to detect protein and mRNA expression of collagen I and α-SMA. Scale bar: 20 μm. (**H**–**J**) After transfection with CD44 siRNA for 36 hours, PMCs were cultured with or without recombinant versican (1 μg/mL) for 24 hours. Then PMCs were harvested for Western blotting (**H** and **I**) and qRT-PCR (**J**) to detect protein and mRNA expression of fibronectin, collagen I, and α-SMA. (**G**, **I**, and **J**) Data are presented as mean ± SEM. Statistical analyses were performed with 1-way ANOVA. *n* = 3, *P < 0.05, **P < 0.01, ***P < 0.001, ****P < 0.0001.

Versican binds to cell surface proteins (receptors) to activate downstream signal pathways. CD44, integrin β1, and EGFR are reported to interact with versican (27–30). To investigate whether these cell surface receptors contribute to versican mediating matrix viscoelasticity and pleural fibrosis, primary rat PMCs were cultured in hydrogels with different degrees of viscoelasticity for 24 hours. As shown in Figure 5, A and B, CD44 increased and no obvious changes in integrin β1 or EGFR were observed in PMCs cultured in high viscoelastic hydrogels. Next, expression of CD44, integrin β1, and EGFR in response to recombinant versican was measured in primary human PMCs. Recombinant versican increased CD44 and did not change integrin β1or EGFR (Figure 5, C–E). Furthermore, inhibition of integrin β1 and EGFR by siRNA did not change the fibrotic process in versican-treated primary rat PMCs (Supplemental Figure 7).

To confirm the role of CD44 in versican-mediated pleural fibrosis, knocking down CD44 was done with CD44 siRNA in primary rat PMCs (Supplemental Figure 8). To directly study the effect of viscoelasticity in fibrosis, primary rat PMCs were cultured in hydrogels with different levels of viscoelasticity. In high-viscoelasticity gels, the expression of MesoMT markers collagen I and α-SMA increased, and both were attenuated by CD44 siRNA (Figure 5, F and G). Moreover, qRT-PCR and Western blotting showed that versican induced mRNA and protein expression of fibronectin, collagen I, and α-SMA. The expression of fibronectin, collagen I, and α-SMA were prevented by CD44 siRNA (Figure 5, H–J).

**Figure 6:**
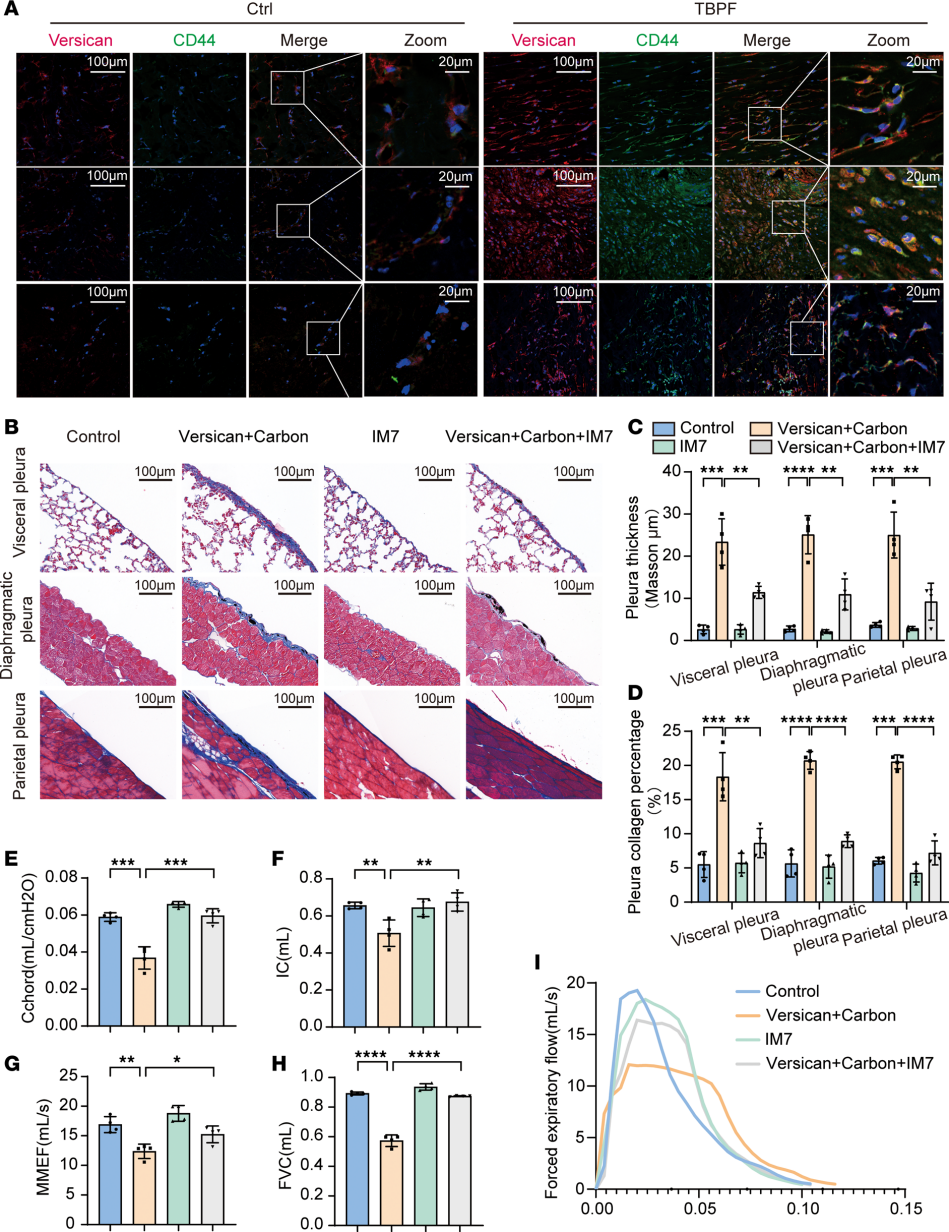
Anti-mouse CD44 neutralizing antibody (IM7) alleviated versican-mediated pleural fibrosis in vivo. (**A**) Representative results for immunostaining of versican and CD44 in pleura from patients and controls. Scale bars: 100 μm. (**B**–**J**) The mouse pleural fibrosis model was induced by intrapleural injections of recombinant versican proteins (150 μg/kg) plus carbon particles (0.1 mg/mouse). IM7 was administrated by intrapleural injection at a dose of 100 μg at days 4, 7, and 10. After lung function testing, all mice were euthanized at day 21, and then pleura were taken for analysis. (**B**) Representative images of Masson’s trichrome staining of visceral pleura from lung sections, parietal pleura from chest wall, and diaphragm sections. Scale bar: 100 μm. (**C**) Changes in pleural thickness. (**D**) Changes in collagen percentages of pleura. (**E**–**I**) Changes in lung function test. Data are presented as mean ± SEM. Statistical analyses were performed with 1-way ANOVA. *n* = 4, **P < 0.01, ***P < 0.001, ****P < 0.0001.

To further confirm the role of CD44 in versican-mediated pleural fibrosis, in vivo experiments were performed. In clinical samples, CD44^hi^ was expressed with versican in patients with pleural fibrotic tissue compared with control tissue (Figure 6A). Recombinant versican proteins plus carbon were intrapleurally injected in mice to develop models. As shown in Figure 6, B–I, versican proteins plus carbon increased pleural thickness in visceral, parietal, and diaphragmatic pleura and decreased lung function metrics including chord compliance, inspiratory capacity, inspiratory capacity, forced vital capacity, and forced expiratory flow. Anti-mouse CD44 neutralizing antibody (IM7) attenuated these changes, which indicated that IM7 prevented pleural fibrosis.

To define whether versican and CD44 had a common effect in pleural fibrosis that resulted from different etiological factors, a mouse pleural fibrosis model was induced by intrapleural injections of Bacillus Calmette-Guérin (BCG) or tuberculous pleural effusion (TBPE). Immunofluorescence analysis demonstrated that versican expression was upregulated in TBPE- and BCG-induced models (Supplemental Figure 9). BCG and TBPE increased pleural thickness in visceral, parietal, and diaphragmatic pleura (Supplemental Figure 10, A–F) and decreased lung function metrics including chord compliance, inspiratory capacity, mean mid-expiratory flow, forced vital capacity, and forced expiratory flow (Supplemental Figure 10, G–P). Versican shRNA or IM7 attenuated these changes, which indicated that versican and CD44 had a common effect in pleural fibrosis. These data provide solid evidence that versican-induced high viscoelasticity mediated pleural fibrosis through CD44.

### Versican/CD44 increased Smad4 through deubiquitination by ubiquitin-specific peptidase 10 in PMCs.

**Figure 7:**
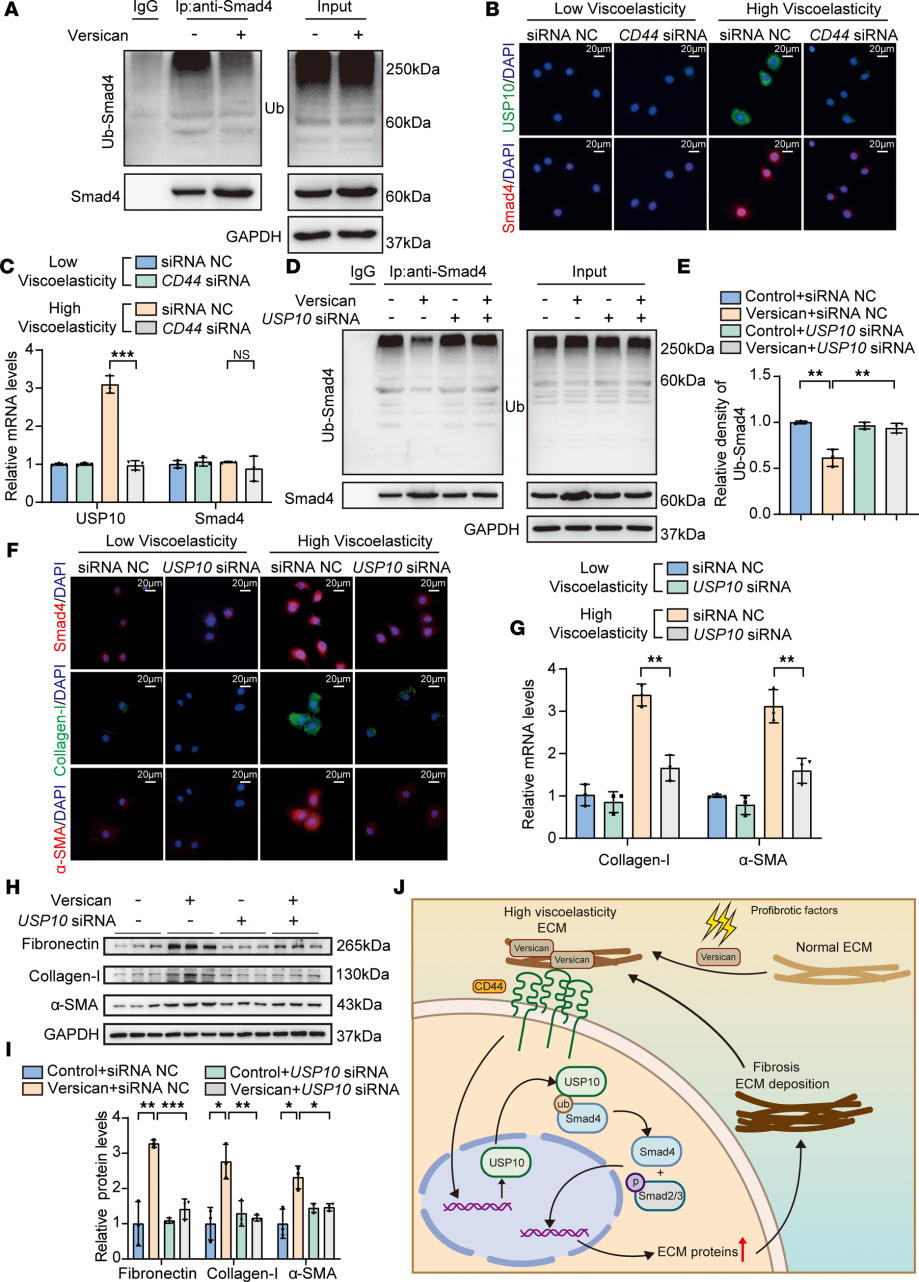
Versican reinforced deubiquitination of Smad4 by recruiting USP10 in PMCs. (**A**) Ubiquitination of Smad4 was detected by IP in PMCs. (**B** and **C**) After transfection with *CD44* siRNA for 36 hours, PMCs were cultured in low- or high-viscoelasticity hydrogels for 48 hours. Then PMCs were harvested for immunostaining (**B**) and qRT-PCR (**C**) to detect protein and mRNA levels of collagen I and α-SMA. (**D** and **E**) After transfection with *USP10* siRNA2 for 36 hours, PMCs were cultured with or without recombinant versican (1 μg/mL) for 24 hours; ubiquitination of Smad4 was detected by IP. (**F** and **G**) After transfection with *USP10* siRNA for 36 hours, PMCs were cultured in low- or high-viscoelasticity hydrogels for 48 hours. Then PMCs were harvested for immunostaining (**F**) and qRT-PCR (**G**) to detect protein and mRNA expression of Smad4, collagen I, and α-SMA. (**H** and **I**) After transfection with control and *USP10* siRNA for 36 hours, PMCs were cultured with or without recombinant versican (1 μg/mL) for 24 hours. Then PMCs were harvested for Western blotting to detect protein expression of fibronectin, collagen I, and α-SMA. (**J**) Schematic illustration of the mechanisms of versican in PMCs. Data are presented as mean ± SEM. Statistical analyses were performed with 1-way ANOVA. *n* = 3, *P < 0.05, **P < 0.01, ***P < 0.001, ****P < 0.0001.

To further explore the downstream signal pathway of versican and CD44 in the pathogenesis of pleural fibrosis, the downstream pathway of TGF-β signal Smad2/3/4 expression was assessed in primary rat PMCs. Interestingly, versican increased Smad4 protein rather than Smad2/3 (Supplemental Figure 11, A and B) without a change in the Smad4 mRNA level (Supplemental Figure 11C). However, Western blotting showed that versican increased phosphorylation of Smad2/3 in the nucleus (Supplemental Figure 11, D–F). These results suggest that versican facilitated translocation of phosphorylated Smad2/3 into the nucleus through Smad4 rather than increasing the total protein level of Smad2/3 in the cells. Ubiquitination of Smad4 has been found to regulate fibrosis; therefore, we investigated the effect of versican on ubiquitination of Smad4 in primary rat PMCs. As shown in Figure 7A, recombinant rat versican protein suppressed ubiquitination and degradation of Smad4, which led to the aggregation of Smad4 in primary rat PMCs. Next, ubiquitin-specific peptidase 10 (USP10) was detected. The confocal images demonstrated that USP10 and Smad4 were colocalized in primary rat PMCs (Supplemental Figure 12A), and USP10 directly interacted with Smad4 in primary rat PMCs (Supplemental Figure 12B). Immunostaining analysis showed that high viscoelasticity increased the protein expression of USP10 and Smad4 in primary rat PMCs (Figure 7B). High viscoelasticity increasing USP10 was attenuated by CD44 siRNA without a change in Smad4 mRNA (Figure 7C).

To further confirm that USP10 mediated posttranslational modification of Smad4, USP10 siRNA was used in PMCs (Supplemental Figure 13). Versican inhibited deubiquitination of Smad4 and increased the Smad4 level, and these changes were prevented by USP10 siRNA (Figure 7, D and E). More importantly, recombinant versican proteins and high viscoelasticity induced upregulation of collagen I and α-SMA mRNAs and proteins, and USP10 siRNA attenuated their mRNA and protein levels (Figure 7, F–I, and Supplemental Figure 14).

To demonstrate traction-independent CD44 signaling as the mechanistic basis of the observed effects, we conducted key experiments in the versican-hydrogels system in the presence of the myosin II inhibitor blebbistatin to suppress actomyosin contractility. Under this condition, the specific activation of CD44 and the associated downstream cellular effects induced by the high-viscoelastic matrix persisted (Supplemental Figure 15). This finding demonstrated that the observed CD44 signaling is independent of the integrin/actomyosin traction force pathway. To directly distinguish between the “HA-CD44 ligation” and “altered matrix physical properties” hypotheses, we constructed versican and an HA binding–deficient versican mutant (Supplemental Figure 16A). Co-IP assays confirmed that this deficient mutant cannot bind to CD44 (Supplemental Figure 16B). However, it still promoted cellular fibrosis (Supplemental Figure 16, C–E), indicating that fibrosis is driven not by HA-CD44 ligation but by altered matrix viscoelasticity.

Collectively, as shown in Figure 7J, this study demonstrated that versican/CD44 increased Smad4 through USP10 deubiquitination in PMCs, which contributed to versican-mediated fibrogenesis in pleura.

## Discussion

In this study, we found that ECM from fibrotic tuberculous pleura promoted MesoMT in PMCs, and excessive versican protein in ECM played an important role in MesoMT. Analysis of human tissue samples demonstrated that a high level of versican was associated with pleural fibrosis in patients. Next, results from animal models showed that excessive versican in fibrotic pleural ECM enhanced ECM viscoelasticity and consequently promoted pleural fibrosis. In vitro, recombinant versican and high viscoelasticity mediated MesoMT through the CD44/USP10/Smad4 mechanotransductive pathway in PMCs. Inhibition of versican or CD44 was demonstrated to attenuate pleural fibrosis in mice.

Versican, which belongs to the chondroitin sulfate proteoglycan family, is mainly found in ECM (31). Reduced versican expression seems to impair cartilage development and endochondral ossification, resulting in dwarfism (32, 33). Studies involving versican gene knockout have demonstrated its importance in cardiac development, as the lack of versican resulted in multiple structural defects, including ventricular septal defect, abnormal cardiac looping, outflow tract constriction, and loss of cardiac jelly (34, 35). To the best of our knowledge, few studies have addressed the role of versican in pleural fibrosis. In our study, we found that versican protein was upregulated in pleura of patients with TBPF, and recombinant versican proteins promoted MesoMT in PMCs, demonstrating that versican is likely an active contributor to pleural fibrosis, rather than merely a consequence of fibrotic tissue remodeling. Versican is recognized to modulate cell behavior through interactions with cell surface receptors, including CD44, EGFR, and integrin β1 (27–30). Our findings demonstrated that versican increased viscoelasticity and promoted pleural fibrosis by CD44, rather than integrin β1 or EGFR.

To elucidate how versican mediates PMC-MesoMT and fibrosis, 4 methods (bleomycin plus carbon particles, recombinant versican plus carbon particles, tuberculous pleural effusion, and BCG induction) were employed to establish pleural fibrosis models. Within these experimental models, blockade of versican and CD44 attenuated pleural fibrosis. The data indicated that versican/CD44 exerted a general effect in pleural fibrosis induced by various etiological factors. Currently, only limited therapeutic options exist for managing pleural fibrosis (36); thus, versican/CD44 may represent a promising therapeutic target for pleural fibrosis. Furthermore, ECM deposition in idiopathic pulmonary fibrosis (IPF) is a similar process as in pleural fibrosis (37, 38), so it is conceivable that versican/CD44 may provide valuable insights into the pathogenesis of IPF.

Our study also demonstrated that versican promoted pleural fibrosis via enhancing the viscoelasticity of pleural tissue. Previous research has revealed that during adipose conversion in cells, the viscoelastic properties of the medium changed due to the presence of versican (39). Furthermore, versican contributed to hydrated viscoelastic ECM, which accommodated cyclic mechanical forces on vessels. At the same time, accumulation of versican in ECM decreased the residence time for cell-associated receptors that participated in ECM formation, including elastin binding protein, which consequently impaired elastic fiber formation and affected cell proliferation and viscoelasticity of the intima (25, 26). Versican affects ECM assembly and controls elastic fiber formation, which plays a critical role in ECM restructuring in cardiovascular diseases (32). In the current study, we confirmed that versican gene *VCAN* knockout reversed bleomycin-induced increases of pleural viscoelasticity in mice. Moreover, a high level of versican and viscoelasticity promoted MesoMT in PMCs, indicating that versican enhanced pleura viscoelasticity and promoted pleural fibrosis.

The Smad pathway is a signaling cascade in which TGF-β is recognized by TβR II, which possesses an intracellular kinase domain (40, 41). Downstream components of the Smad cascade, particularly Smad2 and Smad3, are regarded as key effectors of TGF-β signaling in both tissue fibrosis and tumor development (42, 43). Acting as a convergent node for TGF-β and bone morphogenetic protein (BMP) signaling, Smad4 enables the nuclear entry of Smad2/3 complexes upon TGF-β exposure and Smad1/5/8 complexes upon BMP exposure (44). In vitro, depletion of Smad4 in mesangial cells suppresses collagen I promoter (45). In our study, activation of the TGFβ1/Smad4 axis by versican and increased viscoelasticity was the key point in the signaling pathway in PMCs leading to fibrosis. Additionally, we found that versican and increased viscoelasticity upregulated the protein level rather than the transcription of Smad4. A previous study reported that USP10 was involved in the deubiquitination of Smad4 and promoted metastasis in hepatocellular carcinoma. (46). We found that USP10-mediated deubiquitination of Smad4 led to increased Smad4 levels in cells.

In conclusion, our study demonstrated that versican, a component of ECM, promoted pleural fibrosis. Inhibition of versican improved pleural fibrosis and lung function in mice. These findings provide strong evidence supporting the clinical relevance of versican and viscoelasticity as a potential therapy for pleural fibrosis. The promise of therapeutic strategies targeting versican to inhibit fibrosis calls for future comprehensive research and clinical investigation.

## Methods

### Sex as a biological variable.

Our study examined men and women, as well as male and female animals, and similar findings are reported for both sexes.

### Reagents and antibodies.

Recombinant human TGF-β1 protein was purchased from R&D Systems. Recombinant rat versican protein was sourced from Abmart (RKRB81754); the control protein contained a GAPDH fragment that was also from Abmart (RKRB93290). Anti-mouse CD44 antibody (IM7) was sourced from MedChemExpress (MCE; HY-P99126). Carbon particles were from Mitsubishi Chemical Corporation. Bleomycin was obtained from Haerbin Laibotong Pharmaceutical Co. Ltd. The antibodies used in the experiments were as follows: anti-VCAN antibody (T58111, Abmart), anti-VCAN antibody (A20278, ABclonal), anti-EGFR antibody (A11082, ABclonal), anti-ITGB1 antibody (A2217, ABclonal), anti-SMAD4 antibody (A5657, ABclonal), anti-SMAD4 antibody (A19116, ABclonal), anti-USP10 antibody (CL488-67917, Proteintech), anti-USP10 antibody (19374-1-AP, Proteintech), anti-CD44 antibody (15675-1-AP, Proteintech), anti–Wilms tumor 1 antibody (ab89901, Abcam), anti-p-smad2/3 antibody (8828, Cell Signaling Technology), anti-t-smad2/3 antibody (8685, Cell Signaling Technology), anti-GAPDH antibody (60004-1, Proteintech), anti-collagen I antibody (67288-1-Ig, Proteintech), anti-fibronectin antibody (15613-1-AP, Proteintech), anti-α-SMA antibody (14395-1-AP, Proteintech), and IgG antibody (10283-1-AP, Proteintech).

### Human samples.

Pleural tissue samples from patients with TBPF and normal pleural tissue resected from patients with lung adenocarcinoma were obtained with informed consent. The diagnostic criteria for tuberculous pleurisy included the identification of *Mycobacterium tuberculosis* in pleural fluid or the presence of caseating granulomas in closed pleural biopsy specimens in the absence of evidence of any other granulomatous diseases. Tuberculous pleurisy was diagnosed according to Light’s criteria. Definitive diagnosis of tuberculous pleurisy was based on the detection of *Mycobacterium tuberculosis* in pleural fluid or pleural tissue or demonstration of caseating granulomas in pleural biopsy. Clinical data of patients with TBPF and control group participants are provided in Supplemental Table 1.

### Pleura ECM extraction.

Pleural tissue from patients was washed with PBS, embedded in optimal cutting temperature compound, and frozen at –80°C. Pleura were cut transversely into 100 μm fragments using a cryostat and immersed in 2% Triton X-100 and 20 mM EDTA solution in double-distilled water on a horizontal shaker overnight at room temperature. The remaining pleural ECM was washed 5 times with PBS and subsequently placed in 10% penicillin-streptomycin amphotericin B solution (Gibco) for sterilization. Prior to matrix administration to culture, fragments were washed with serum-free medium and homogenized using gentle MACS M tubes (Miltenyi Biotec). The protein concentration of the supernatant (enriched in soluble ECM components) was determined using a BCA assay (Thermo Fisher Scientific). The ECM supernatant was diluted to a final working concentration of 20 μg/cm^2^ in sterile PBS. The coating solution was evenly added to cover the entire growth surface of the culture dish/plate. The dishes were then incubated overnight at 4°C under sterile conditions to allow for optimal adsorption of ECM proteins onto the polystyrene surface. The following day, the coating solution was carefully aspirated. To remove any unbound material and salts, the coated surfaces were gently rinsed twice with sterile PBS. The dishes were used immediately for cell seeding.

Primary PMCs were cultured in 6-well plates until confluent and maintained for an additional 24 hours to allow ECM deposition. Cells were removed by incubation with a solution of 0.5% Triton X-100 and 20 mM NH_4_OH in PBS for 10 minutes at room temperature with gentle agitation. The plates were then rinsed extensively with PBS (at least 5 times) to remove all cellular debris. The resulting acellular ECM matrices were used for subsequent cell reseeding experiments.

### Isolation and identification of primary human PMCs.

Fresh pleural effusion was first filtered through sterile gauze to remove debris, followed by centrifugation at 1,500*g* for 6 minutes at 4°C to collect the cell pellet. The pellet was resuspended in RPMI-1640 or DMEM/F-12 medium supplemented with 10% FBS and 1% penicillin-streptomycin and seeded onto uncoated culture flasks. To enhance purity, cells were cultured under low-serum conditions (2% FBS) with the addition of EGF (10 ng/mL) to promote mesothelial cell proliferation. This isolation and culture protocol was consistent with our previously published methods (47, 48).

### ELISA.

Pleural effusion samples were collected and centrifuged at 1,000*g* for 10 minutes at 4°C to remove cells and debris. The supernatants were stored at –80°C until analysis. The concentration of versican in pleural effusion was measured using a commercial ELISA kit (Bioswamp Biotechnology Co., Ltd.; SHM11073) according to the manufacturer’s instructions. Absorbance was measured at 450 nm using a microplate reader, and versican levels were calculated based on the standard curve.

### Pleural fibrosis models.

C57BL/6J mice were obtained from Hubei BIONT Biological Technology Co. (Wuhan, China). Mice (males, 6–8 weeks of age, 18–20 g) were intrapleurally injected with bleomycin (20 U/kg) and carbon particles (0.1 mg/mouse), recombinant versican proteins (150 μg/kg) plus carbon particles (0.1 mg/mouse), BCG, or tuberculous pleural effusion (5 μL/g) in the right pleural cavity, 3–5 mm to the right of the sternum at the fifth intercostal space; control mice received an equal volume of saline. Lentivirus expressing shRNA directed against *VCAN* (gene of versican) or scrambled sequence shRNA was administrated by intrapleural injection at a dose of 2 × 10^6^ transduction units (TU) on days 4, 7, and 10. IM7 was administrated by intrapleural injection at a dose of 100 μg on days 4, 7, and 10. All mice were euthanized on day 21 after lung function measurements. Mice were housed in specific pathogen–free facilities at Animal Center of Tongji Medical College with a 12-hour light/12-hour dark cycle. BCG (China strain) was a gift from the Laboratory of Xionglin Fan at Tongji Medical College, Huazhong University of Science and Technology (49). All experimental procedures were approved by the IACUC of Tongji Medical College, Huazhong University of Science and Technology.

### Rheometry analysis of the human and mouse pleura.

Pleural samples were prepared using a 20 mm diameter punch. The height of the slices ranged from 1 to 2 mm in the uncompressed state. The samples were kept hydrated during the experiments with standard RPMI-1640 medium. Parallel plate shear rheometry was performed on the Discovery HR-20 rheometer (TA Instruments) at room temperature. For all measurements, the upper plate was initially lowered to touch the sample, and 0.01 N of nominal initial force (~300 Pa) was applied to ensure adhesive contact of the sample with the plates. Measurements were taken first with a dynamic time sweep test (2% constant strain, oscillation frequency 1 radian s^−1^, measurements taken for 600 seconds), and then stress relaxation (10% initial strain, measurements taken for 600 seconds). Frequency sweep tests were conducted in oscillatory shear mode under steady-state conditions within the linear viscoelastic region, as determined by prior amplitude sweep tests. The angular frequency (ω) was logarithmically varied from 0.1 to 100 rad/s at a constant strain amplitude. Complex viscosity (η*) was automatically calculated using the instrument’s software.

### Preparing alginate with different viscoelasticity in cell culture.

According to the manufacturer, low–molecular mass, ultrapure sodium alginate (Provona UP VLVG, Sigma-Aldrich, 42000501) with a molecular mass of less than 75 kDa was used for fast-relaxing substrates. For slow-relaxing substrates, sodium alginate with a molecular mass of more than 200 kDa was used (Provona UP MVG, Sigma-Aldrich, 42000101). Alginate was treated with activated charcoal, dialyzed against deionized water for 3–4 days (molecular mass cutoff 3,500 Da), sterile-filtered, lyophilized, and then reconstituted to 3.5 wt% in serum-free DMEM (Gibco). The use of low/high–molecular mass alginate resulted in high/low-viscoelasticity hydrogels (50).

### Mouse lung function assessment.

To assess mouse lung function, mice were anesthetized with sodium pentobarbital solution for endotracheal intubation and connection to a Forced Maneuvers system (e.g., CRFM100, EMMS). Respiration was monitored under spontaneous breathing using whole-body plethysmography and respiratory rate sensors connected to a data acquisition system. Lung function parameters measured were lung compliance, elasticity, resistance including chord compliance, inspiratory capacity, mean mid-expiratory flow, and forced vital capacity. The experimental procedures strictly adhered to animal experimental ethics standards, and statistical methods were employed for data analysis to compare lung function differences among different groups or posttreatment conditions.

### Cell culture.

Rat primary PMCs were isolated from rat pleura with protease as in our previous studies (10, 36). In brief, the whole thorax was isolated under sterile conditions after 1 mg/mL protease from streptomyces griseus (Sigma-Aldrich) in 5 mL RPMI-1640 medium was injected into the thoracic cavity and then digested at 4°C overnight. The cells were harvested and centrifuged at 120 × *g* for 5 minutes. The spun-down cells were resuspended in epithelial cell medium-animal (ScienCell) and incubated for 7 days and then replaced with RPMI-1640 containing 20% FBS. Other treatments were the same as with Met-5A cells. To confirm the phenotype of the rat primary PMCs, the isolated cells were incubated with antibodies against Wilms tumor 1 (dilution 1:50) and calretinin (dilution 1:50) at 4°C overnight. The Cy3- (AS007) or FITC-labeled (AS001) secondary antibody IgG (ABclonal) was added and incubated for 30 minutes. The nucleus was stained for DAPI for 10 minutes in the dark. Labeled cells were examined using a fluorescence microscope (Olympus FV500).

### Immunoblotting and IP.

As previously described, the cultured cells underwent washing, trypsinization, and centrifugation to obtain pure cells. Then, cells were lysed with RIPA lysis buffer (Sevicebio) and protease inhibitors. The protein concentration was measured with a BCA protein determination kit (Thermo Fisher Scientific, 23225). Afterward, an equal amount of protein was loaded and separated on a 10% SDS-PAGE gel and then transferred to a PVDF membrane (Merck Millipore). After blocking in 5% skimmed milk, the membrane was incubated at 4°C overnight with the primary antibody, followed by HRP-conjugated goat anti-rabbit secondary antibody (1:10,000; RRID: AB_2099233; Cell Signaling Technology), incubated together, and left at room temperature for 1 hour. The primary antibody dilutions used in this study were as follows: versican (1:1,000), fibronectin (1:1,000), collagen I (1:1,000), α-SMA (1:1,000), CD44 (1:1,000), ITGB1 (1:1,000), EGFR (1:1,000), Smad4 (1:1,000), USP10 (1:1,000), p-Smad2/3 (1:500), t-Smad2/3 (1:500), GAPDH (1:5,000). Secondary antibody dilutions were 1:10,000 for GAPDH and 1:5,000 for the others.

The co-IP test was performed according to the manufacturer’s protocol (Protein A/G Magnetic Beads, HYK0202, MCE). Briefly, PMCs were lysed in octyl-D-glucoside (2%) buffer. Afterward, the supernatant protein (500 mg) and anti-USP10 antibody were spun overnight at 4°C, and then spun with Protein A/G Magnetic Beads at 4°C for 4 hours. Subsequently, the sample was washed 4 times in Tris-buffer. The protein was eluted with Laemmli sample buffer, and then the sample was boiled at 100°C for 10 minutes to detect USP10 and Smad4 by Western blotting. Reverse verification was performed as well. The results are expressed as the ratio between the expression intensity of Smad4 and the expression intensity of immunoprecipitated USP10.

### Transfection of siRNA.

The versican-specific siRNA and corresponding scrambled siRNA were purchased from Ambion (sequence information in Supplemental Table 3). Primary PMCs were seeded in 12-well plates 24 hours before transfection. Transient transfection was performed using Lipofectamine 3000 (Invitrogen) when cells reached 60% confluency in RPMI-1640 medium without FBS and antibiotics. Transfection efficiency was evaluated 24 hours after transfection using qRT-PCR or Western blotting. Three siRNAs targeting versican were tested. CD44 siRNA, ITGB1 siRNA, EGFR siRNA, and USP10 siRNA were used in the same way as the versican siRNA. All RNA interference sequences designed in this study are listed in Supplemental Table 2.

### Quantitative PCR analysis.

Total cellular RNA was extracted using TRIzol reagent. RNA levels were determined by qRT-PCR. PCR was run under the following conditions after the total RNA was reverse transcribed: 95°C for 30 seconds, 40 cycles of 95°C for 10 seconds, and 60°C for 20 seconds. The results were expressed as 2^−ΔΔCT^ using GAPDH as a reference. All RNA sequences designed in this study are listed in Supplemental Table 3.

### IHC.

Slides were incubated with anti-versican antibody for 1 hour, and then processed according to the ABC Peroxidase Standard Staining kit (Thermo Fisher Scientific) for 30 minutes. The slides were stained with 3,3′-diaminobenzidine (Abcam) for 5 seconds to 5 minutes and counterstained with hematoxylin (Thermo Fisher Scientific) for 45 seconds. The images were scanned using the Pannoramic SCAN system (3DHISTECH).

### Immunofluorescence staining.

PMCs were fixed with 4% paraformaldehyde for 20 minutes, permeabilized with 0.5% Triton X-100 for 20 minutes, and then blocked with 5% BSA in PBS-Tween for 1 hour. Subsequently, PMCs were diluted with antibody in 5% BSA at 4°C overnight. DAPI was used to counterstain the nuclei. For human and mouse tissues, specific tissues were fixed in PBS containing 4% paraformaldehyde for 48 hours, embedded in paraffin, sectioned, and stained as previously described. Fluorescence images were captured using a confocal microscope.

### Tandem mass tag.

Total proteins were extracted from fresh pleura and protein quality was assessed. Qualified samples were subjected to trypsin digestion and tandem mass tag labeling separately. The labeled peptides were then mixed in equal amounts to form a composite. After desalting, the composite underwent fractionation using high-pH reversed-phase high-performance liquid chromatography (RP-HPLC), producing 12 distinct peptide fractions. Each fraction was subjected to nano-HPLC reversed-phase chromatography and mass spectrometric detection. Protein identification and quantitative analysis were performed using PD/MaxQuant search software. Quantitative results were standardized and subjected to statistical analysis to identify differentially expressed proteins for subsequent bioinformatics analysis. The mass spectrometry proteomics data have been deposited to the ProteomeXchange Consortium via the iProX partner repository with the dataset identifier PXD073100.

### Statistics.

Data were analyzed using GraphPad Prism software. For comparisons between 2 groups, an unpaired, 2-tailed Student’s *t* test was employed. Multiple-group comparisons were conducted using 1-way ANOVA. Gene and protein expression data were normalized using GAPDH as a reference, with stability verified across experimental conditions. Statistical significance was set at *P* less than 0.05. All in vitro experiments were conducted using cells derived from multiple independent donors. For all experiments, a specimen from a single donor or cells from a single animal were counted as *n* = 1 in the statistical analysis. All experiments were independently replicated at least 3 times, and results are expressed as mean ± SD, consistently reported throughout the study.

### Study approval.

Clinical sample collection and related experiments in this study were approved by the Human Ethics Committee of Union Hospital, Tongji Medical College, Huazhong University of Science and Technology (approval 2022 IEC 043). All samples from patients were obtained with written informed consent. All animal-related experimental procedures were approved by the IACUC of Tongji Medical College, Huazhong University of Science and Technology (approval 20220304).

### Data availability.

All data and materials are included in the manuscript and the Supporting Data Values file. The mass spectrometry proteomics data have been deposited to the ProteomeXchange Consortium via the iProX partner repository with the dataset identifier PXD073100. Further information and requests for resources and reagents should be directed to and will be fulfilled by the corresponding author.

## Author contributions

ZHJ, HY, and WLM conceptualized the study. ZHJ, XLC, QL, PPC, and LQZ contributed to the methodology. ZHJ, XLC, QL, PPC, LQZ, SYY, SHH, CYL, and HDZ conducted the investigation. ZHJ, XLH, HY, and WLM contributed to visualization. HY, WLM, LX, and MW acquired funding. ZHJ, XLH, LML, LJS, FY, LX, FX, XW, MW, XD, HY, and WLM contributed to data analysis and logical organization. ZHJ, HY, and WLM wrote the original draft of the manuscript. WLM, HY, XD, and ZHJ contributed to writing and review of the manuscript. ZHJ participated in the conception of this study, thus, ZHJ was listed before XLH in the order of co-first authors.

## Conflict of interest

The authors have declared that no conflict of interest exists.

## Funding support

- National Natural Science Foundation of China (82270111 to WLM).
- National Natural Science Foundation of China (82270075 and 82470100 to HY).
- National Natural Science Foundation of China (82070098 to LX).
- National Natural Science Foundation of China (82200081 to MW).

## Supplementary Material

Supplemental data

Unedited blot and gel images

Supporting data values

## Version 1 04/23/2026

In-Press Preview

## Version 2 06/22/2026

Electronic publication
