# Supplementary material for: Versican regulating viscoelasticity drives pleural fibrosis via mechanotransductive signaling
Source: JCI Insight. 2026 Apr 23;11(12):e199507. doi: 10.1172/jci.insight.199507 (PMC13313549; doi:10.1172/jci.insight.199507)
Supplement: Supplemental data [file jciinsight-11-199507-s082.pdf]

Online supplementary materials

Title: Versican regulating viscoelasticity drives pleural fibrosis via mechanotransductive signaling

Authors: Zi-Heng Jia *et al*

Contents:

Tables:

Table S1. Demographics of patients recruited in the study

Table S2. Sequences of siRNA

Table S3 Primers used for qRT-PCR

Figures:

Figure S1. Identification of primary human pleural mesothelial cells (HPMCs)

Figure S2. Fibrotic pleural ECM mediated pro-fibrogenic phenotypes in PMCs

Figure S3. Versican overexpressed in human pleural fibrosis

Figure S4. Excessive versican was primarily derived from PMCs

Figure S5. Validation of lentivirus efficiency in mouse models

Figure S6. Versican mediated pleural fibrosis in PMCs *in vitro*

Figure S7. ITGB1 and EGFR did not affect versican-mediated pleural fibrosis

Figure S8. Validation of CD44 siRNA efficiency in PMCs

Figure S9. Versican expression up-regulated in TBPE and BCG-induced models

Figure S10. Versican shRNA and IM7 alleviated TBPE or BCG-induced pleural fibrosis in mice.

Figure S11. Versican facilitated translocation of phosphorylated Smad2/3 into the nucleus through Smad4

Figure S12. USP10 directly interacted with Smad4 in PMCs

Figure S13. Validation of USP10 siRNA efficiency in PMCs

Figure S14. Versican encouraged pleural fibrosis in USP10 dependent manner

Figure S15. CD44 signaling was independent of traction

Figure S16. HA-binding-deficient versican mutant

Table S1. Demographics of patients recruited in the study

Subject	Inpatient ID	Age	Sex	Clinical diagnosis
Subject1	362***6	70	M	Lung adenocarcinoma
Subject2	361***8	62	M	Lung adenocarcinoma
Subject3	362***7	34	F	Lung adenocarcinoma
Subject4	182***0	66	F	Lung adenocarcinoma
Subject5	363***2	62	M	Lung adenocarcinoma
Subject6	363***3	53	M	Lung adenocarcinoma
Subject7	347***5	61	M	Lung adenocarcinoma
Subject8	362***8	74	F	Lung adenocarcinoma
Subject9	139***5	61	F	Lung adenocarcinoma
Subject10	363***8	58	F	Lung adenocarcinoma
Subject11	319***4	68	F	Lung adenocarcinoma
Subject12	358***7	64	F	Lung adenocarcinoma
Subject13	403***2	69	M	Tuberculous pleurisy
Subject14	403***4	54	M	Tuberculous pleurisy
Subject15	403***8	41	M	Tuberculous pleurisy
Subject16	403***4	52	M	Tuberculous pleurisy
Subject17	403***7	50	M	Tuberculous pleurisy
Subject18	403***3	67	M	Tuberculous pleurisy
Subject19	403***2	20	M	Tuberculous pleurisy
Subject20	404***3	39	M	Tuberculous pleurisy
Subject21	404***1	39	M	Tuberculous pleurisy
Subject22	404***7	52	F	Tuberculous pleurisy
Subject23	404***1	51	M	Tuberculous pleurisy
Subject24	404***5	72	M	Tuberculous pleurisy
Subject25	404***4	21	M	Tuberculous pleurisy
Subject26	404***6	54	F	Tuberculous pleurisy
Subject27	404***6	75	M	Tuberculous pleurisy
Subject28	404***1	19	M	Tuberculous pleurisy
Subject29	404***4	26	M	Tuberculous pleurisy
Subject30	404***4	53	M	Tuberculous pleurisy
Subject31	404***3	34	F	Tuberculous pleurisy
Subject32	404***3	64	M	Tuberculous pleurisy
Subject33	404***9	35	M	Tuberculous pleurisy
Subject34	404***9	52	F	Tuberculous pleurisy
Subject35	404***0	32	M	Tuberculous pleurisy
Subject36	404***9	72	F	Tuberculous pleurisy
Subject37	404***6	26	M	Tuberculous pleurisy
Subject38	404***5	31	F	Tuberculous pleurisy
Subject39	404***3	69	M	Tuberculous pleurisy
Subject40	404***8	67	M	Tuberculous pleurisy
Subject41	404***1	62	M	Tuberculous pleurisy

Subject42	404***1	57	M	Tuberculous pleurisy
Subject43	404***7	62	M	Tuberculous pleurisy
Subject44	404***1	18	M	Tuberculous pleurisy
Subject45	404***1	36	M	Tuberculous pleurisy
Subject46	404***5	59	M	Tuberculous pleurisy
Subject47	402***7	71	M	Tuberculous pleurisy
Subject48	402***1	45	M	Tuberculous pleurisy
Subject49	402***8	77	M	Tuberculous pleurisy
Subject50	402***1	68	M	Tuberculous pleurisy
Subject51	402***9	51	M	Tuberculous pleurisy
Subject52	402***6	42	M	Tuberculous pleurisy
Subject53	402***7	76	F	Tuberculous pleurisy
Subject54	402***5	77	M	Tuberculous pleurisy
Subject55	402***4	81	F	Tuberculous pleurisy

Table S2. Sequences of siRNA

Gene	Sequences（5'-3')
VCAN siRNA(rat) -1	GGAGAUGCUAGUACUGAUAUG
	UAUCAGUACUAGCAUCUCCUG
VCAN siRNA(rat) -2	GAAACAGAAUUGUCAACUACC
	UAGUUGACAAUUCUGUUUCGG
VCAN siRNA(rat) -3	CCUUCGAAAUAGACAUAUAUC
	UAUAUGUCUAUUUCGAAGGAA
CD44 siRNA(rat) -1	GAAGGGCGAGUAUAGAACAUU
	UGUUCUAUACUCGCCCUUCUU
CD44 siRNA(rat) -2	GGACCAGUUACCAUAACUAUU
	UAGUUAUGGUAACUGGUCCAU
CD44 siRNA(rat) -3	GAAGUACUACUUCAGACAACC
	UUGUCUGAAGUAGUACUUCUG
USP10 siRNA(rat) -1	CAGCAGAGUUCCAAAGAAUUU
	AUUCUUUGGAACUCUGCUGUU
USP10 siRNA(rat) -2	GGCUGUUCGCAGUGGUCUACC
	UAGACCACUGCGAACAGCCUA
USP10 siRNA(rat) -3	GAGUUAUUGGAGACUGUAACC
	UUACAGUCUCCAAUAACUCUG
EGFR siRNA(rat) -1	CGAAAUUUGUGCUACGCAA
	UUGCGUAGCACAAAUUUCG
EGFR siRNA(rat) -2	CGUAGUUGAUGCUGAUGAA
	UUCAUCAGCAUCAACUACG
EGFR siRNA(rat) -3	AGACUACUAUGAAGUAGAA
	UUCUACUUCAUAGUAGUCU
Itgb1 siRNA(rat) -1	GAGAGAGAAUACAAAUGAA
	UUCAUUUGUAUUCUCUCUC
Itgb1 siRNA(rat) -2	CCACAGAAGUUUACAUUAA
	UUAAUGUAAACUUCUGUGG
Itgb1 siRNA(rat) -3	GAGUAACAAUAAAUUACAA
	UUGUAAUUUAUUGUUACUC

Table S3 Primers used for qRT-PCR

Gene		Human Sequences	Rat Sequences
VCAN	Forward	ACTGAAACTTCCTACGTATGCA	CACTTCCTGCTGGTCACACTCAAG
	Reverse	CTCACAAAGTGCACCAACATAA	GCTTATGTCCTCTGGCTGCTTTCC
CD44	Forward	ACTGCT TATGAAGGAAACTGGAACC	GCAGCAGGGTTGTCGCCATAC
	Reverse	TGCCTGGATTGTGCTTGTAGAATG	CACACCTTGCATTCCAGCTCCTC
ITGB1	Forward	AGATGGGAAACTTGGTGGCATTG	ACTCAGTGAACAGCAACGGTGAAG
	Reverse	CTGGACAAGGTGAGCAATAGAAGG	TCCAAATCAGCAGCAAGGCAAGG
EGFR	Forward	ACAGCATAGACGACACCTTCCTC	CACTACGCCGCCTGCTTCAAG
	Reverse	TGGCTTGGACACTGGAGACTG	ACTGTGCCAAATGCTCCTGAACC
USP10	Forward	CAAGGTGAAGGAAGCGAGGATG	ACGGCTGGCTGCGAATTGATG
	Reverse	CCTGGCGGGTGACGGAAG	ACACGGCGGTAATACAGGAGGTAG
SMAD4	Forward	TGCTGCTGGAATTGGTGTTGATG	CTAATGCCACCAGTACCACCAAC
	Reverse	TCTTTGATGCTCTGTCTTGGGTAATC	AGTGAATCCATTCTGCTGCTGTC
COL1A1	Forward	TAGGGTCTAGACATGTTCAGCTTTGT	CACTGTCCTTGTCGATGGCT
	Reverse	GTGATTGGTGGTGGGATGTCT	GGCAGGCGAGATGGCTTATT
α-SMA	Forward	GAGCGTGGCTATTCCTTCGT	AGCATCCGACCTTGCTAACG
	Reverse	GCCCATCAGGCAACTCGTAA	AGAGTCCAGCACAATACCAGTTG
GAPDH	Forward	TGGCTACAGCAACAGGGTGG	TGCCACTCAGAAGACTGTGG
	Reverse	GGTACATGACAAGGTGCGGCT	TTCAGCTCTGGGATGACCTT
Fibronectin	Forward	GGCGACAGGACGGACATCTTTG	AGGCACAAGGTCCGAGAAGAGG
	Reverse	GGCACAAGGCACCATTGGAATTTC	GGTCAAAGCATGAGTCATCCGTAGG

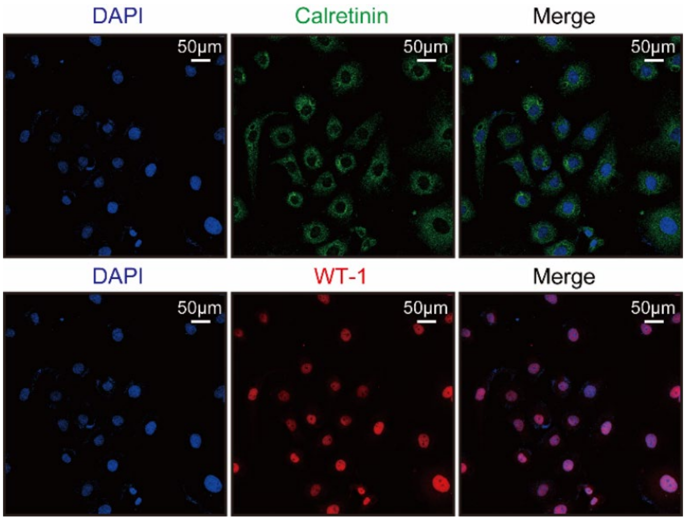

Figure S1. Identification of primary human pleural mesothelial cells (HPMCs). As described in the Methods, primary HPMCs were isolated from pleural effusion. HPMCs were harvested for immunostaining to reveal expression of mesothelial cell markers calretinin (green) and WT-1(red). Scale bars, 50 μm.

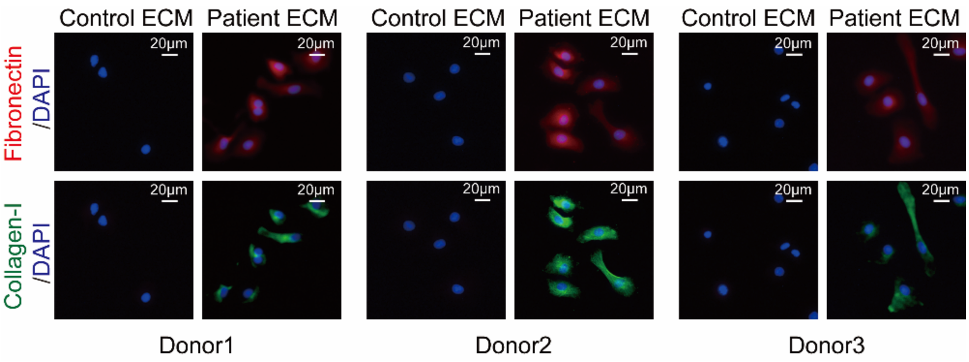

Figure S2. Fibrotic pleural ECM mediated pro-fibrogenic phenotypes in PMCs. Pleura from control subjects and patients were prepared to make ECM as described in the Methods, after which human primary PMCs from three donord were cultured. Immunofluorescence staining of fibronectin and collagen-I was performed in HPMCs. Scale bars, 20 μm.

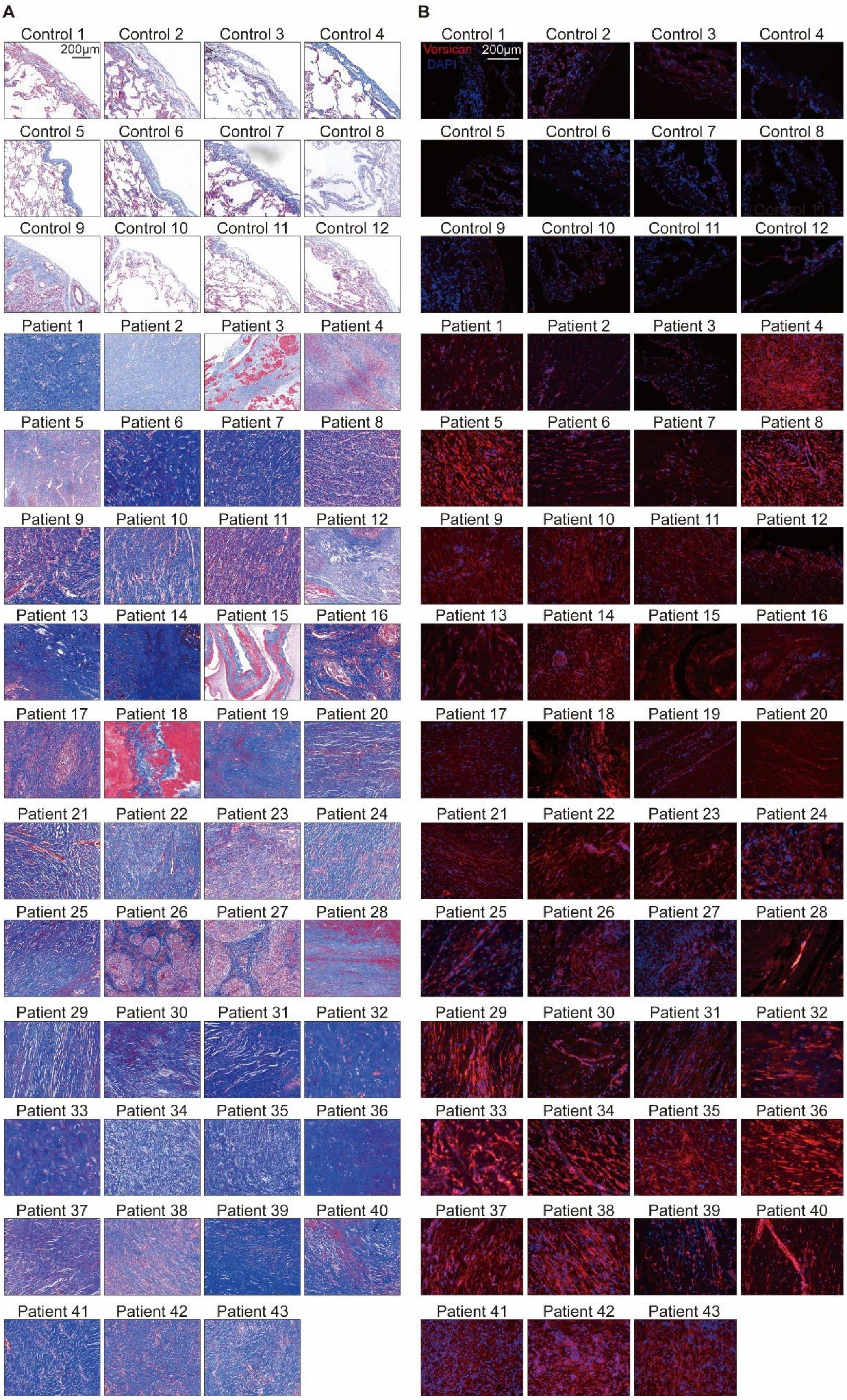

Figure S3. **Versican overexpressed in human pleural fibrosis.** (A) Representative images of

Masson's trichrome staining in pleura from control subjects and TBPF patients. Scale bars, 100 μm. (**B**) Representative images of immunofluorescence staining of versican in pleura from control subjects and TBPF patients. Scale bars, 200 μm.

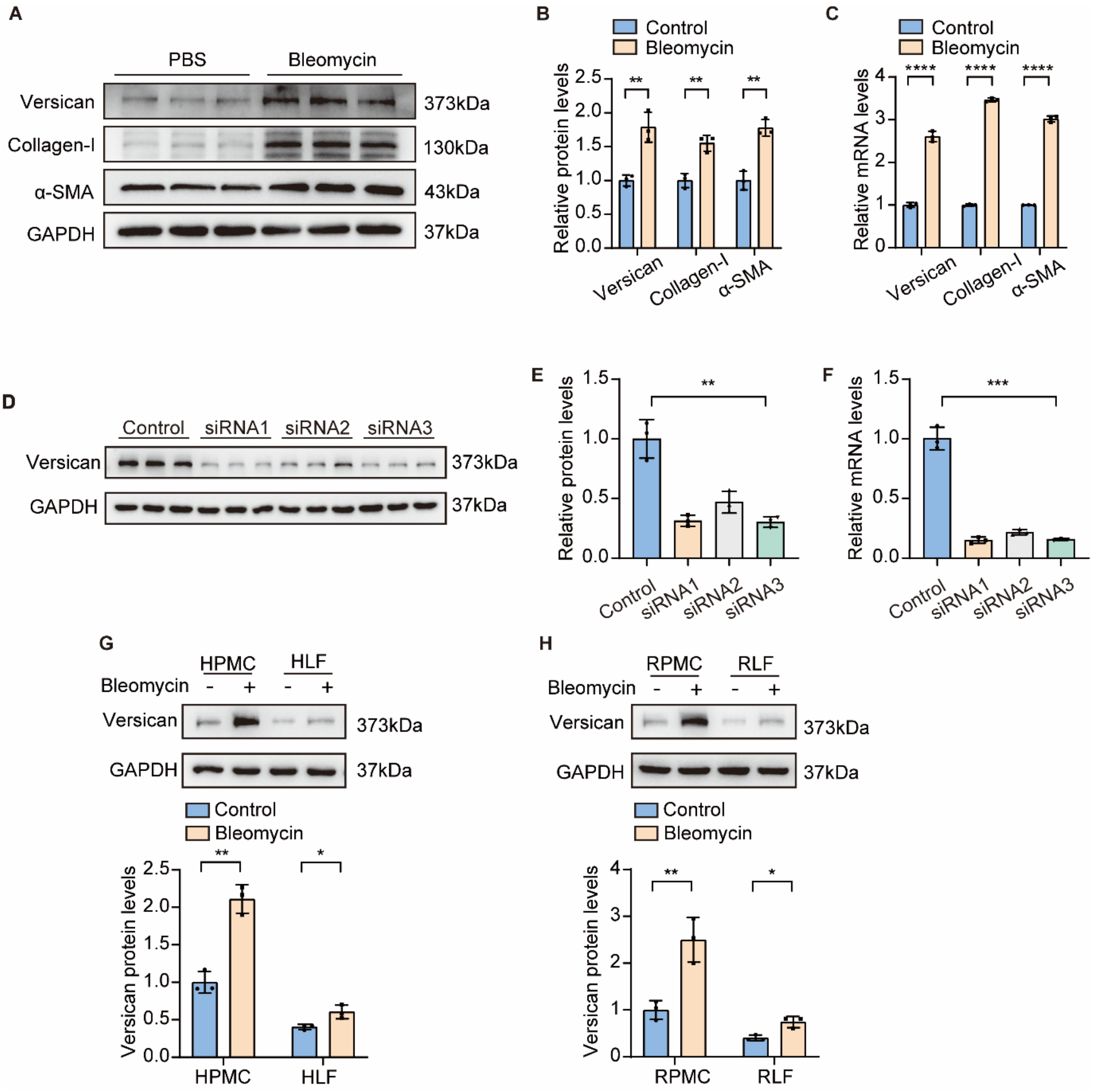

Figure S4. **Excessive versican was primarily derived from PMCs.** (**A-C**) PMCs were incubated with BLEOMYCIN (0.2 μg/ml) for 24 h, after which intracellular protein levels of versican, collagen-I, and α-SMA were measured by Western blotting, mRNA levels of versican, collagen-I, and α-SMA were detected by qRT-PCR at 12 h. (**D-F**) PMCs were transfected with control or siRNAs specific for versican (siRNA1, siRNA2 and siRNA3) for 36 h. The efficiency of siRNA knockdown was assessed by western blotting and qRT-PCR. (**G-H**) Primary human pleural mesothelial cells (HPMC), primary human lung fibroblasts (HLF), primary rat pleural mesothelial cells (RPMC), primary rat lung fibroblast (RLF) were treated

with bleomycin (BLEOMYCIN, 0.2 μg/ml) for 24 h, after which versican protein levels were detected by Western blotting. Results were expressed as mean ± SEM. Statistical significance was determined by unpaired Student's t-tests. n=3. *$P < 0.05$, **$P < 0.01$.

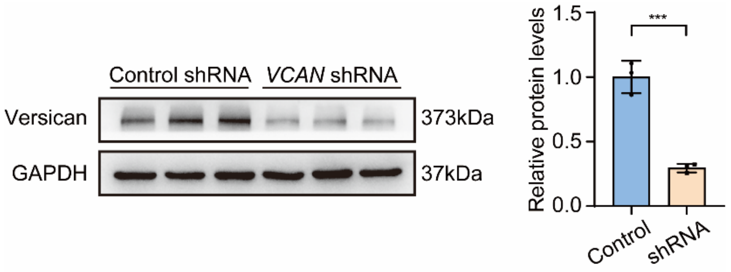

Figure S5. Validation of lentivirus efficiency in mouse models. Lentivirus expressing shRNA directed against versican (VCAN) shRNA or scrambled sequence shRNA were administrated by intrapleural injection at a dose of 2 × 10^6 TU on days 4, 7, and 10. All mice were euthanized at day 21, and then tissues were digested and centrifuged to isolate pleural mesothelial cells. The efficiency of shRNA knockdown was assessed by western blotting, n=3. Data were presented as mean ± SEM. Statistical analysis were performed by unpaired Student's t-tests. n=3, ***P<0.001.

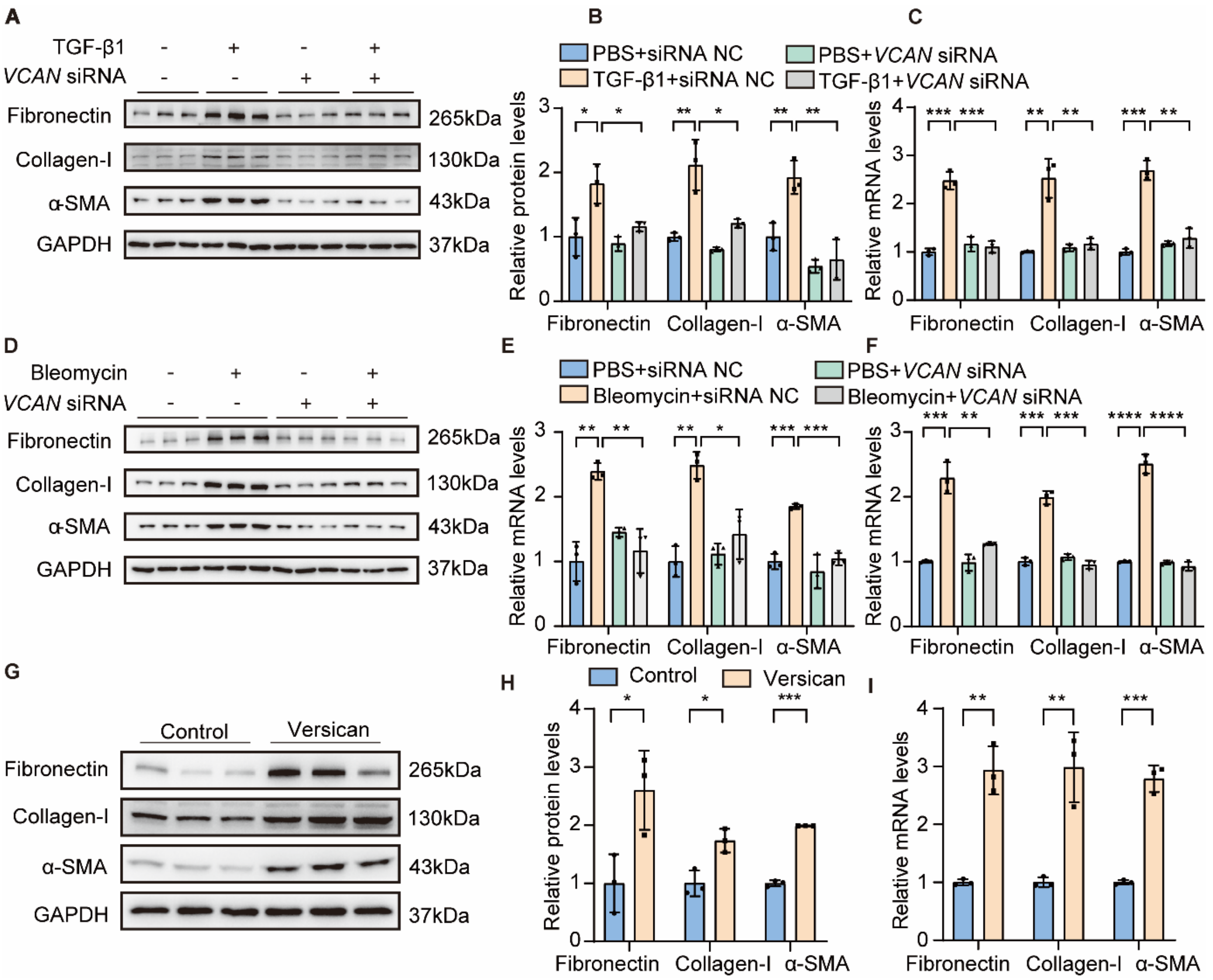

Figure S6. Versican mediated pleural fibrosis in PMCs *in vitro*. (**A-F**) After transfected with siRNA against versican (siRNA3 was selected and used, *VCAN* siRNA) or control siRNA for 36 h, PMCs were cultured with or without TGF-β1 (5 ng/ml) or BLEOMYCIN (0.2 μg/ml) for 24 h, then indicated experiments were performed. PMCs were harvested for western blotting and qRT-PCR to detect protein and mRNA expressions of fibronectin, collagen-I and α-SMA respectively. (**A**, **D**) Images of western blots. (**B**, **E**) Changes of protein levels according to A, D. (**C**, **F**) Changes of mRNA levels. (**G-I**) PMCs were treated by recombinant versican (1 μg/ml), after which fibronectin, collagen-I and α-SMA proteins or mRNA levels were detected by western blotting (**G**, **H**) or qRT-PCR (**I**) respectively. Data were presented as mean ± SEM. (**H, I**) Statistical analysis were performed by unpaired Student's t-tests. (**B, C, E, F**) Statistical analysis were performed by one-way ANOVA. n=3, *P<0.05, **P<0.01, ***P<0.001, ****P < 0.0001.

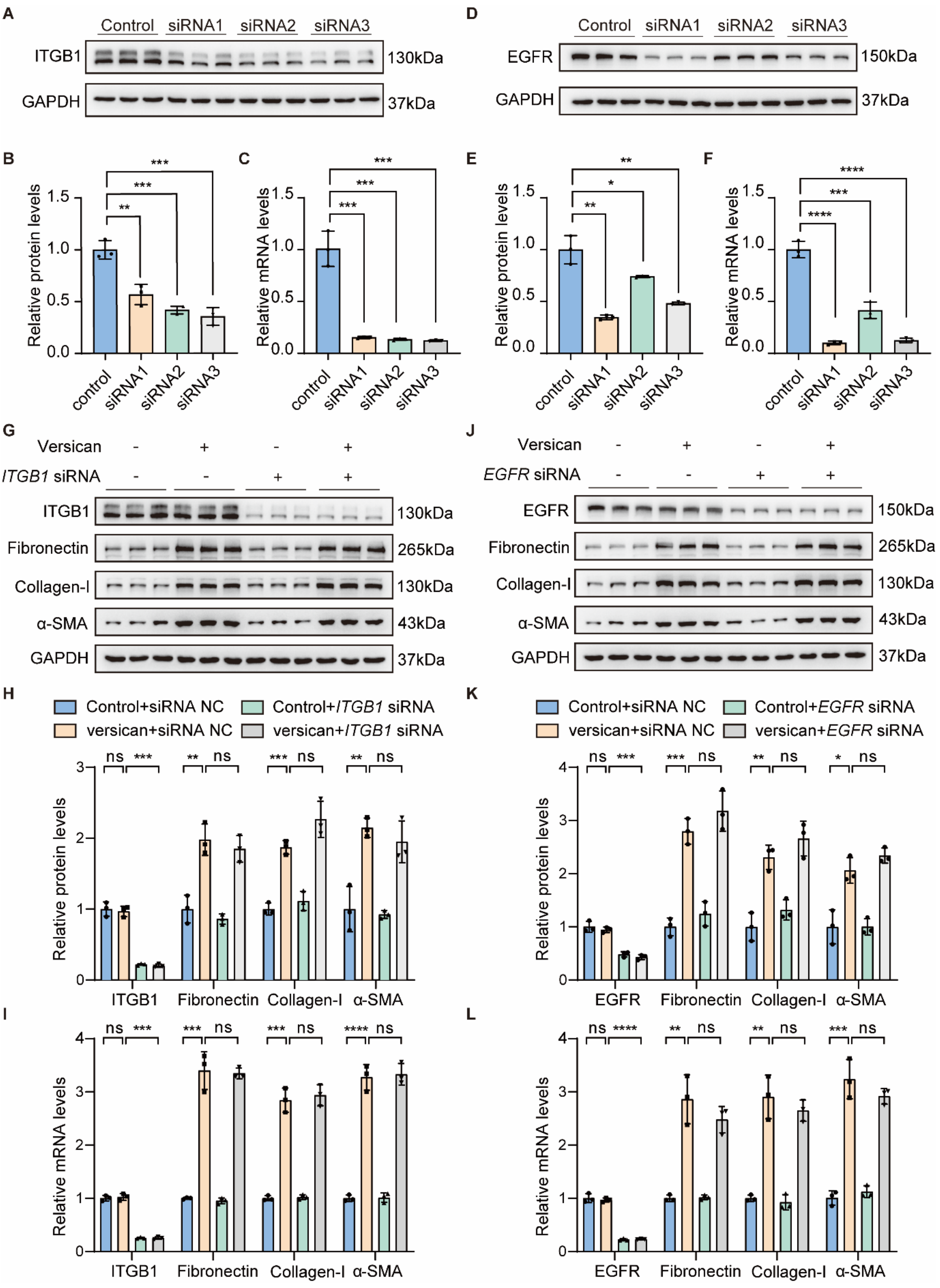

Figure S7. ITGB1 and EGFR did not affect versican-mediated pleural fibrosis. (A-F) PMCs were transfected with siRNAs specific for ITGB1 or EGFR (siRNA1, siRNA2 and

siRNA3) for 48 h. ITGB1/EGFR protein and mRNA knockdown levels were assessed by Western blotting (**A, B, D, E**) and qRT-PCR (**C, F**). Data were presented as mean ± SEM. Statistical analysis were performed by unpaired Student's t-tests. n=3, $^*P<0.05$, $^{**}P<0.01$, $^{***}P<0.001$, $^{****}P < 0.0001$. (**G**-**L**) After transfected with *ITGB1* or *EGFR* siRNA for 36 h, PMCs were cultured with or without recombinant versican (1 μg/ml) for 24 h, then PMCs were harvested for Western blotting (**G, H, J, K**) and qRT-PCR (**I, L**) to detect protein and mRNA expressions of ITGB1/EGFR, fibronectin, collagen-I and α-SMA. Data were presented as mean ± SEM. Statistical analysis were performed by one-way ANOVA. n=3, $^*P<0.05$, $^{**}P<0.01$, $^{***}P<0.001$, $^{****}P < 0.0001$, ns: not significant.

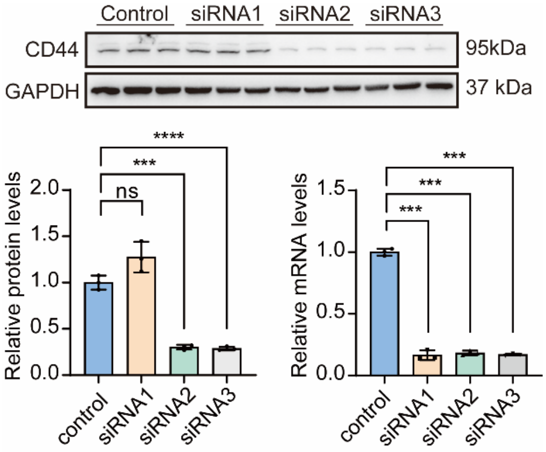

Figure S8. **Validation of CD44 siRNA efficiency in PMCs.** PMCs were transfected with siRNAs specific for CD44 (siRNA1, siRNA2 and siRNA3) for 48 h, the efficiency of knockdown was assessed by Western blotting and qRT-PCR. Data were presented as mean ± SEM. Statistical analysis were performed by unpaired Student's t-tests. n=3, ***P<0.001, ****P < 0.0001, ns: not significant.

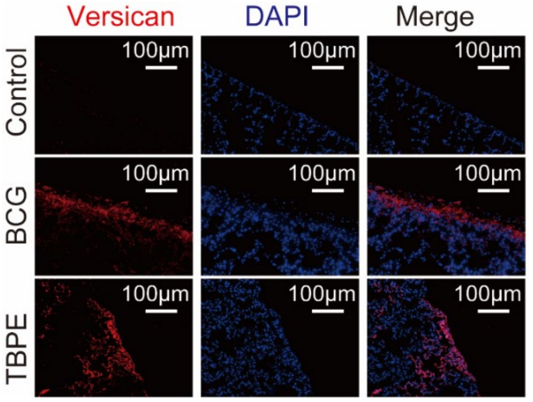

Figure S9. **Versican expression up-regulated in TBPE and BCG-induced models.** The mouse pleural fibrosis model was induced by intrapleural injection of Bacillus Calmette-Guerin (BCG), or tuberculous pleural effusion (TBPE, 5 μl/g). All mice were euthanized at days 21 after lung function measurements. Immunofluorescence staining of versican in visceral pleura. Scale bars, 100 μm.

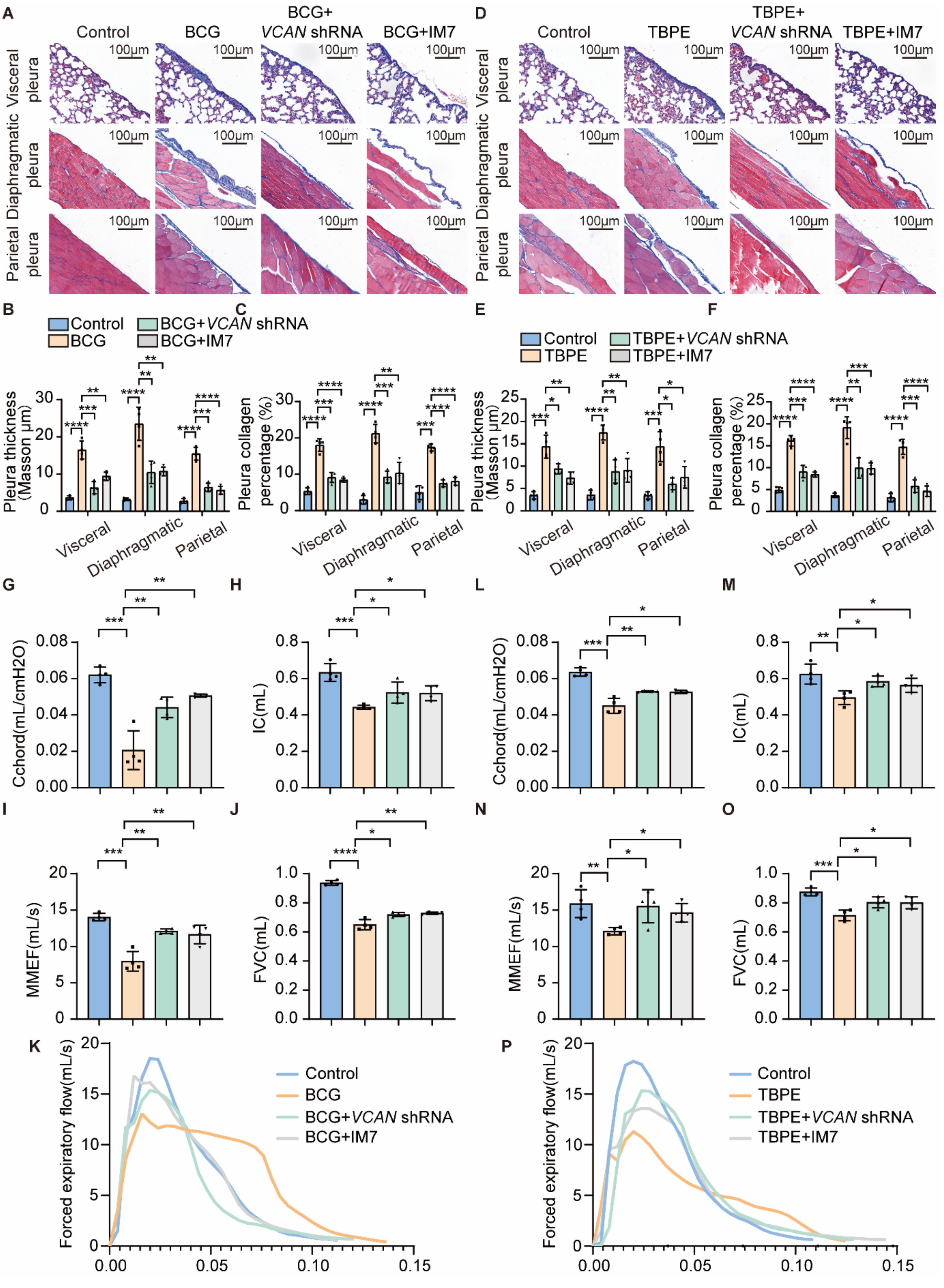

Figure S10. **Versican (*VCAN*) shRNA and IM7 alleviated TBPE or BCG-induced pleural fibrosis in mice.** The mouse pleural fibrosis model was induced by intrapleural injections of

BCG or TBPE. Lentivirus expressing shRNA directed against versican (*VCAN* shRNA) were administrated by intrapleural injection at a dose of 2×10^6 TU on days 4, 7, and 10. IM7 were administrated by intrapleural injection at a dose of 100ug on days 4, 7, and 10. After lung function testing, all mice were euthanized at day 21, and then pleura were taken for analysis. (**A, E**) Representative images of sirius red staining of visceral pleura under polarized light microscopy. Scale bar, 100 μm. (**B, F**) Representative images of Masson's trichrome staining of visceral pleura from lung sections, parietal pleura from chest wall, and diaphragm sections. Scale bar, 100μm. (**C, G**) Changes in pleural thickness. (**D, H**) Changes in collagen percentages of pleura. (**I-R**) Changes in lung function test. Data were presented as mean ± SEM. Statistical analysis were performed by one-way ANOVA. n=4, *P<0.05, **P<0.01, ***P<0.001, ****P < 0.0001.

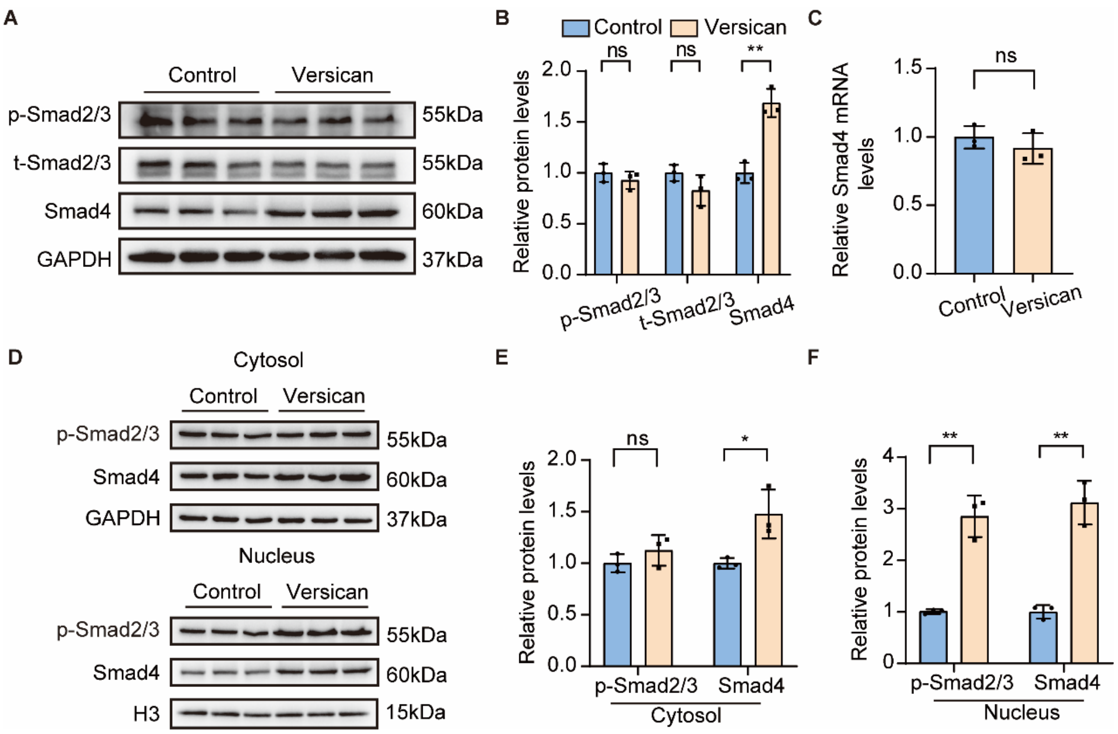

Figure S11. **Versican facilitated translocation of phosphorylated Smad2/3 into the nucleus through Smad4.** (**A**, **B**) Western blotting analysis of p-Smad2/3, t-Smad2/3 and Smad4 expression in PMCs treated by recombinant versican (1 µg/ml) for 12 h. (**C**) RT-PCR analysis of Smad4 expression in PMCs treated by recombinant versican (1 µg/ml) for 12h. (**D**-**F**) Western blotting analysis of p-Smad2/3 and Smad4 expression in nucleus and cytosol. Data were presented as mean ± SEM. Statistical analysis were performed by unpaired Student's t-tests. n=3, *P<0.05, **P<0.01, ns: not significant.

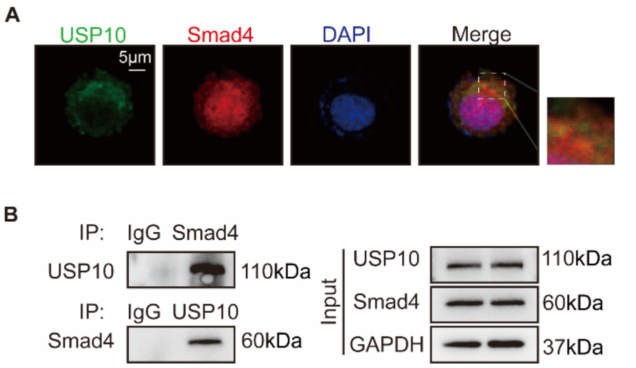

Figure S12. **USP10 directly interacted with Smad4 in PMCs.** (**A**) Representative images for coimmunostaining of USP10 and Smad4 in PMCs. (**B**) PMCs lysates were immunoprecipitated with anti-Smad4 or USP10 antibodies and probed with anti-USP10 or anti-Smad4 antibodies. Scale bars, 5 μm.

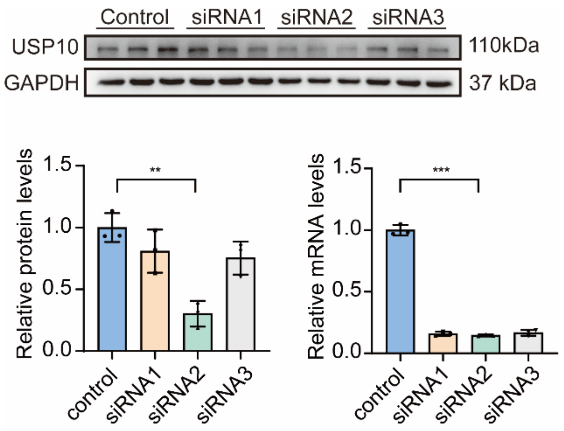

Figure S13. **Validation of USP10 siRNA efficiency in PMCs.** PMCs were transfected with siRNAs specific for USP10 (siRNA1, siRNA2 and siRNA3) for 48 h, the efficiency of knockdown was assessed by Western blotting and qRT-PCR. Data were presented as mean ± SEM. Statistical analysis were performed by unpaired Student's t-tests. n=3, **P<0.01, ***P<0.001.

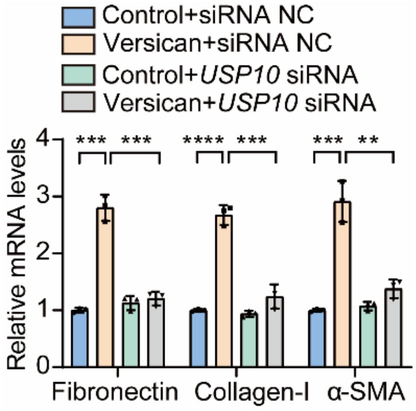

Figure S14.**Versican encouraged pleural fibrosis in USP10 dependent manner.** After transfected with control and *USP10* siRNA for 36 h, PMCs were cultured with or without recombinant versican (1 μg/ml) for 24 h, then PMCs were harvested for qRT-PCR to detect mRNA expressions of fibronectin, collagen-I and α-SMA. Data were presented as mean ± SEM. Statistical analysis were performed by one-way ANOVA. n=3, **P<0.01, ***P<0.001, ****P < 0.0001.

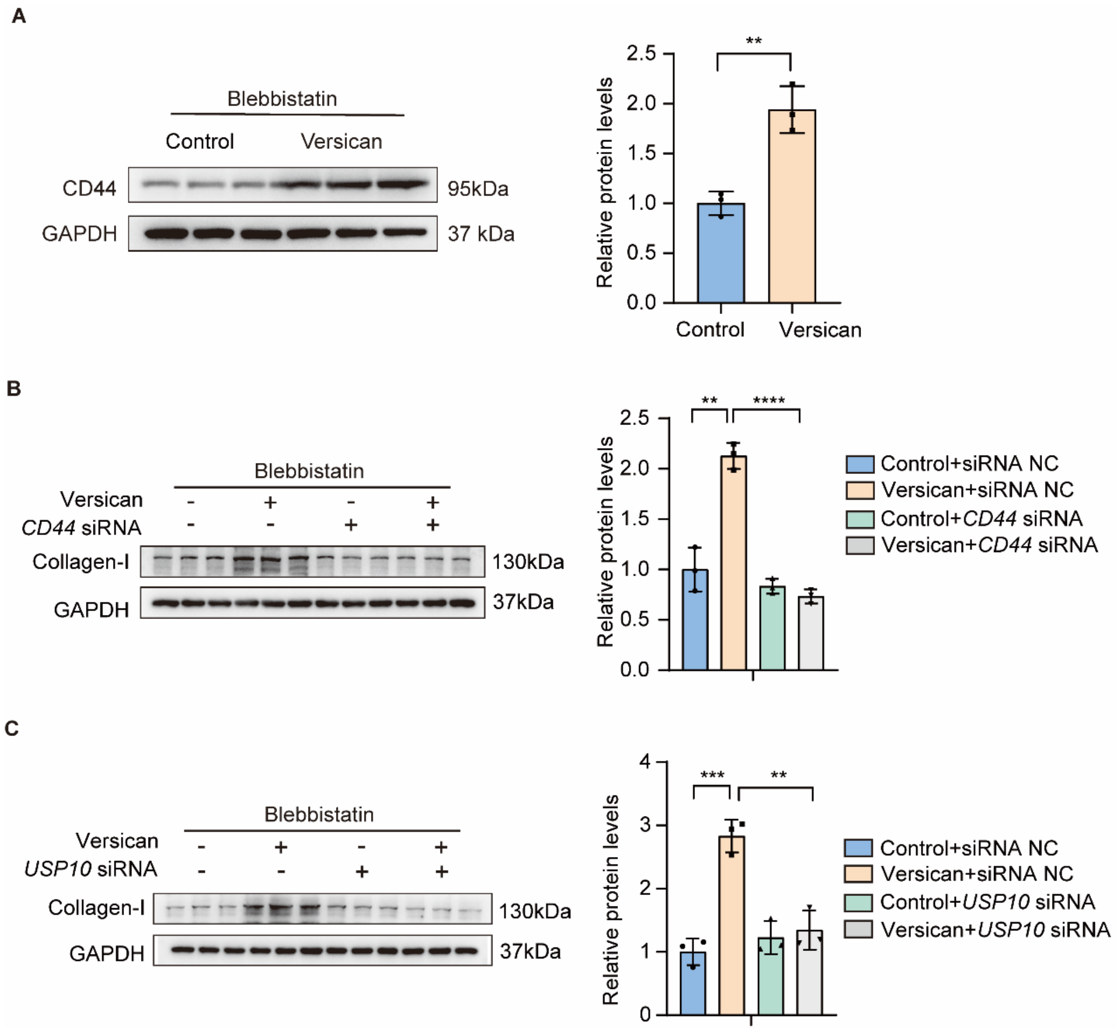

Figure S15. **CD44 signaling was independent of traction.** For the myosin II inhibition test, PMCs were cultured in Medium 1640 with 0.1 vol% blebbistatin (MCE, HY-13813) for 3 days. Then, the cell test was carried out following the approach as described in the Methods. (A) PMCs were treated by recombinant versican (1 μg/ml) for 24 h, after which CD44 was detected by western blotting. Data are presented as mean ± SEM. Statistical analyses were performed by unpaired Student's t-tests. n=3, **P<0.01. (B) After transfected with CD44 siRNA for 36 h, PMCs were cultured with or without recombinant versican (1 μg/ml) for 24 h, then PMCs were harvested for western blotting to detect protein of collagen-I. (C) After transfected with USP10 siRNA for 36 h, PMCs were cultured with or without recombinant versican (1 μg/ml) for 24 h, then PMCs were harvested for western blotting to detect protein of collagen-I. (B, C) Data were presented as mean ± SEM. Statistical analyses were performed by one-way ANOVA. n=3, **P<0.01, ***P<0.001, ****P < 0.0001.

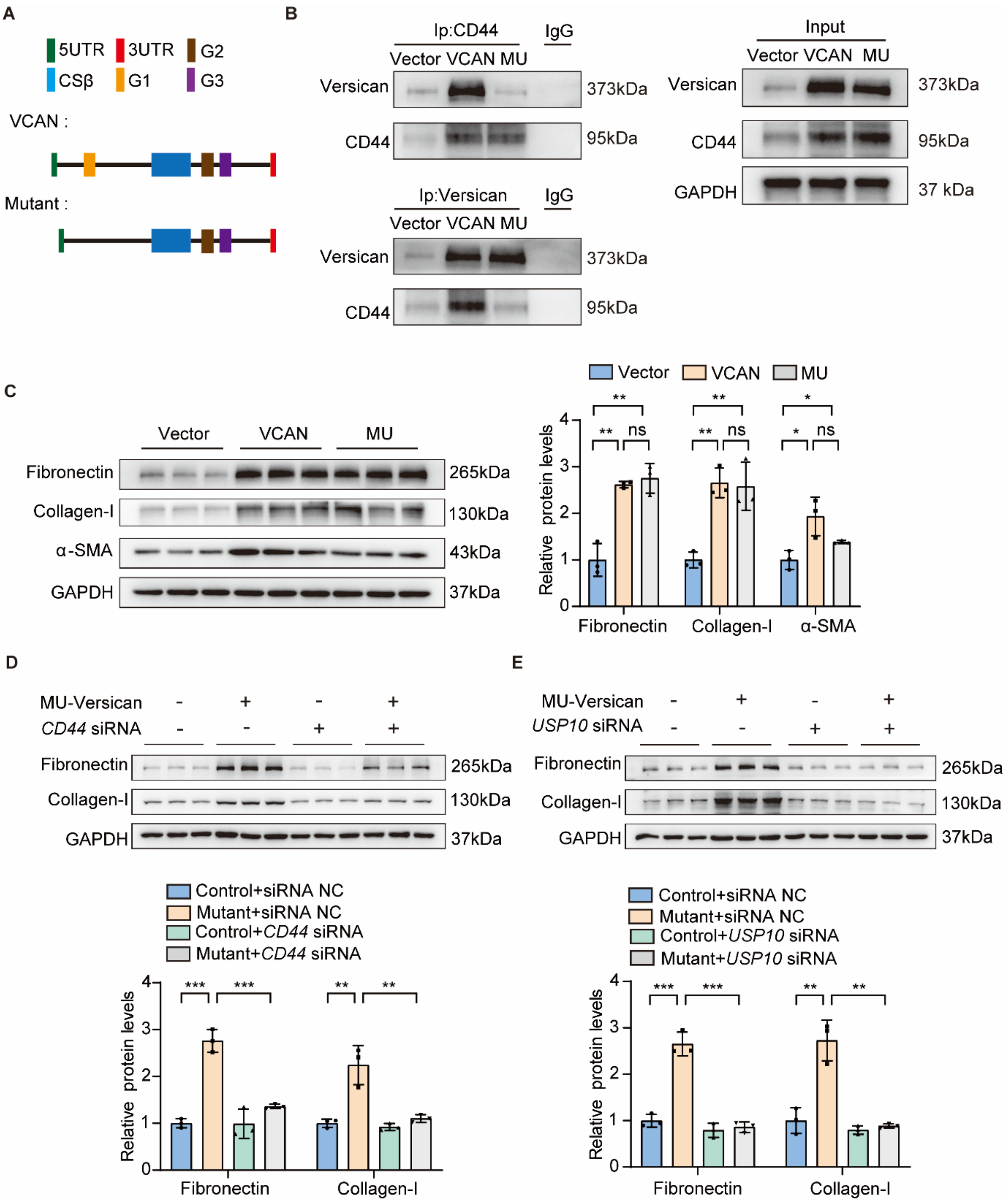

Figure S16. HA-binding-deficient versican mutant. (A) The schematic results showing the mutant plasmids of G1 domain. (B) PMCs lysates were immunoprecipitated with anti-versican or CD44 antibodies and probed with anti-versican or anti-CD44 antibodies. (C) Western blot analysis of the levels of fibronectin, collagen-I and α-SMA in PMCs following plasmid transduction. (D) After transfected with MU-versican5-plasmid and *CD44* siRNA for 36 h,

PMCs were cultured for 24 h, then PMCs were harvested for western blotting to detect protein expressions of fibronectin, collagen-I. (E) After transfected with MU-versican5-plasmid and *USP10* siRNA for 36 h, PMCs were cultured for 24 h, then PMCs were harvested for western blotting to detect protein expressions of fibronectin, collagen-I. Data are presented as mean ± SEM. (C, D, E) Statistical analyses were performed by one-way ANOVA. n=3, *P<0.05, **P<0.01, ***P<0.001.
