# Supplementary material for: Versican regulating viscoelasticity drives pleural fibrosis via mechanotransductive signaling
Source: JCI Insight. 2026 Apr 23;11(12):e199507. doi: 10.1172/jci.insight.199507 (PMC13313549; doi:10.1172/jci.insight.199507)

Full unedited gel for figure 1B

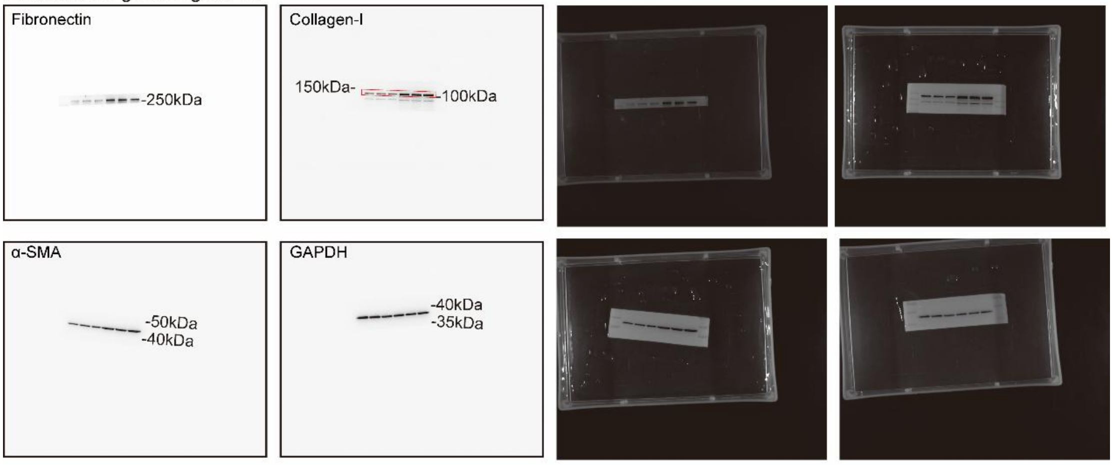

Full unedited gel for figure 1F

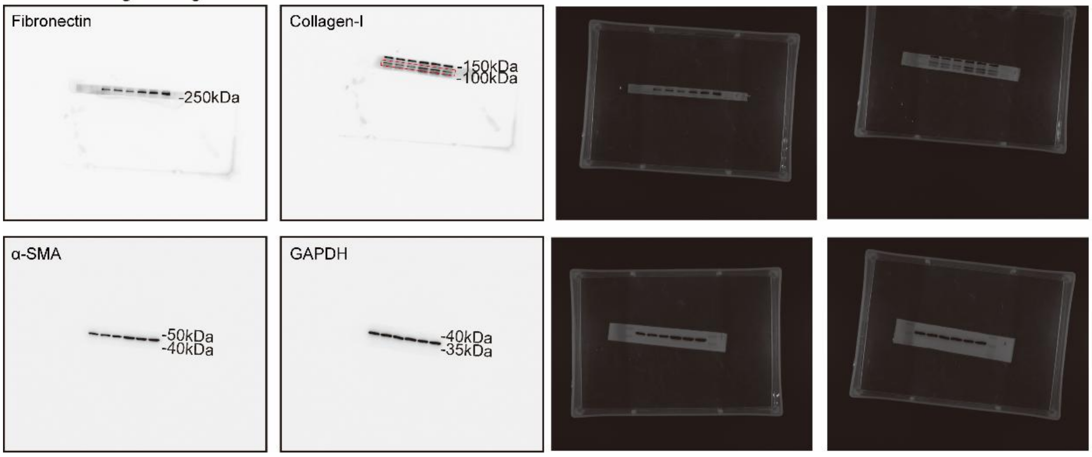

Full unedited gel for figure 2J

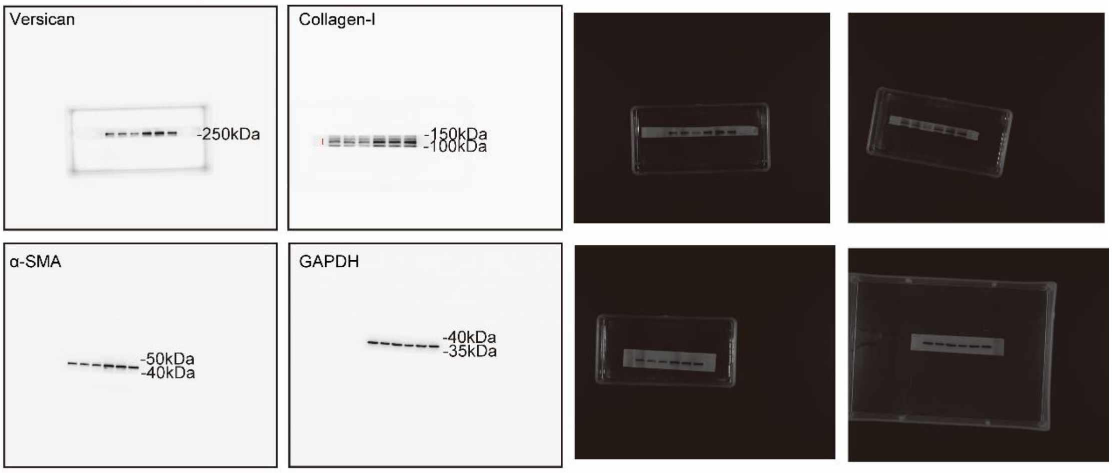

Full unedited gel for figure 2M

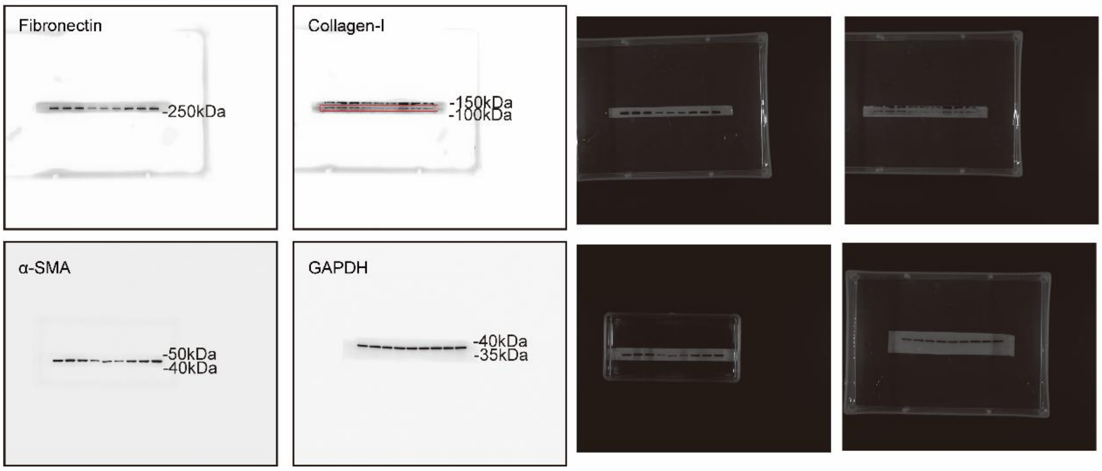

Full unedited gel for figure 5C

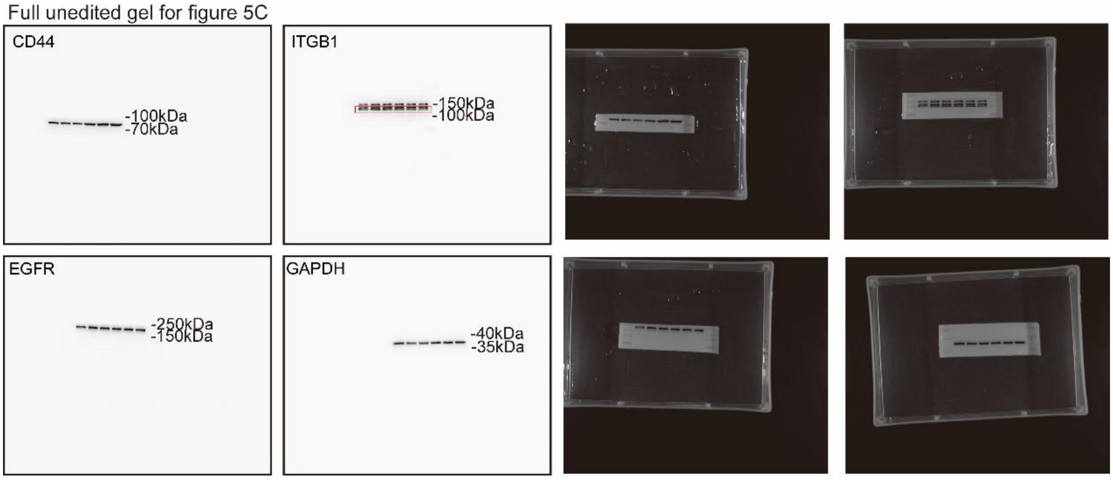

Full unedited gel for figure 5H

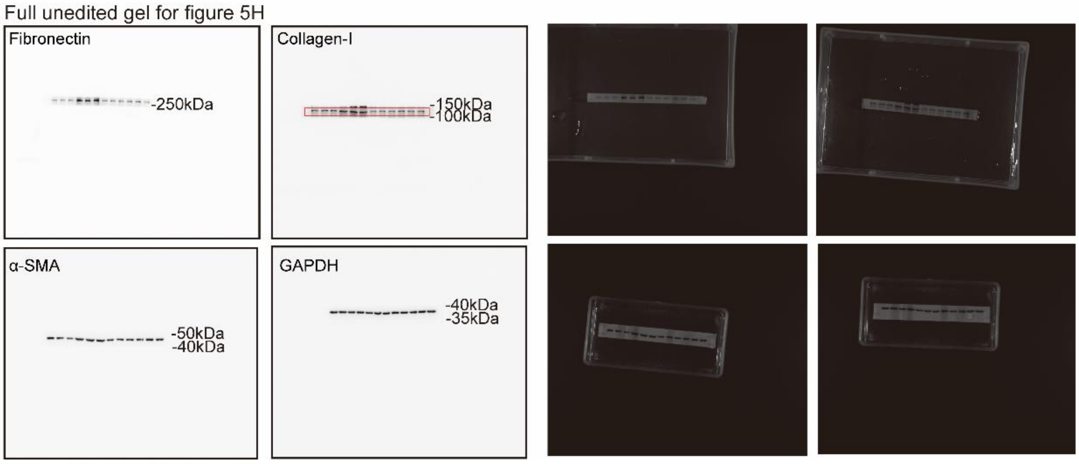

Full unedited gel for figure 7A

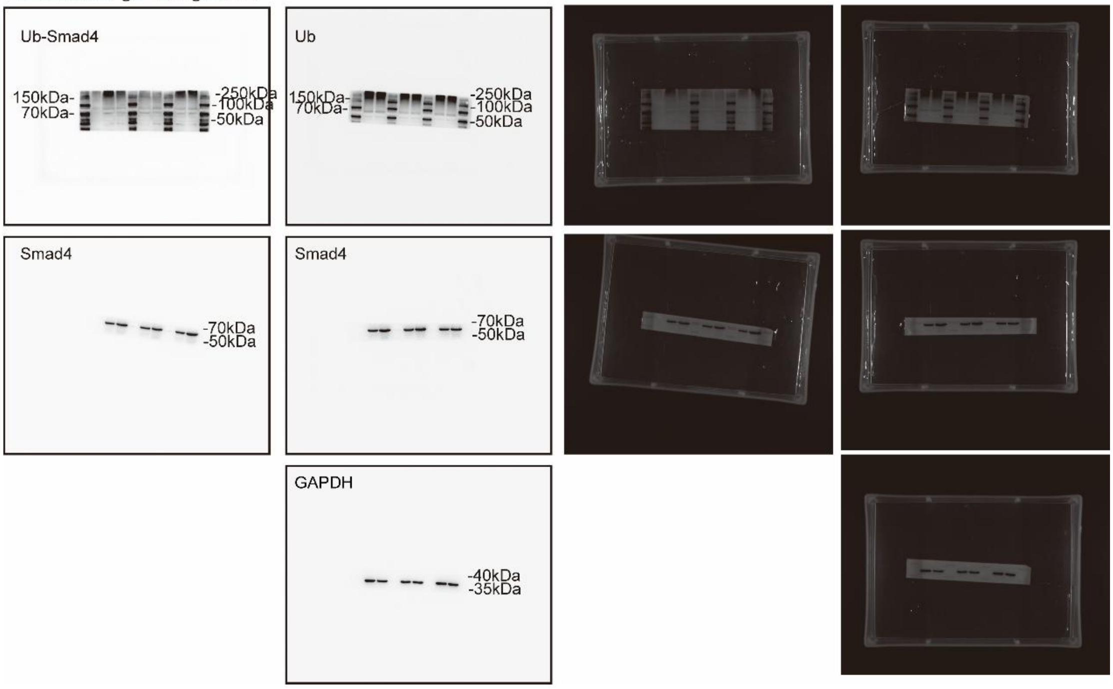

Full unedited gel for figure 7D

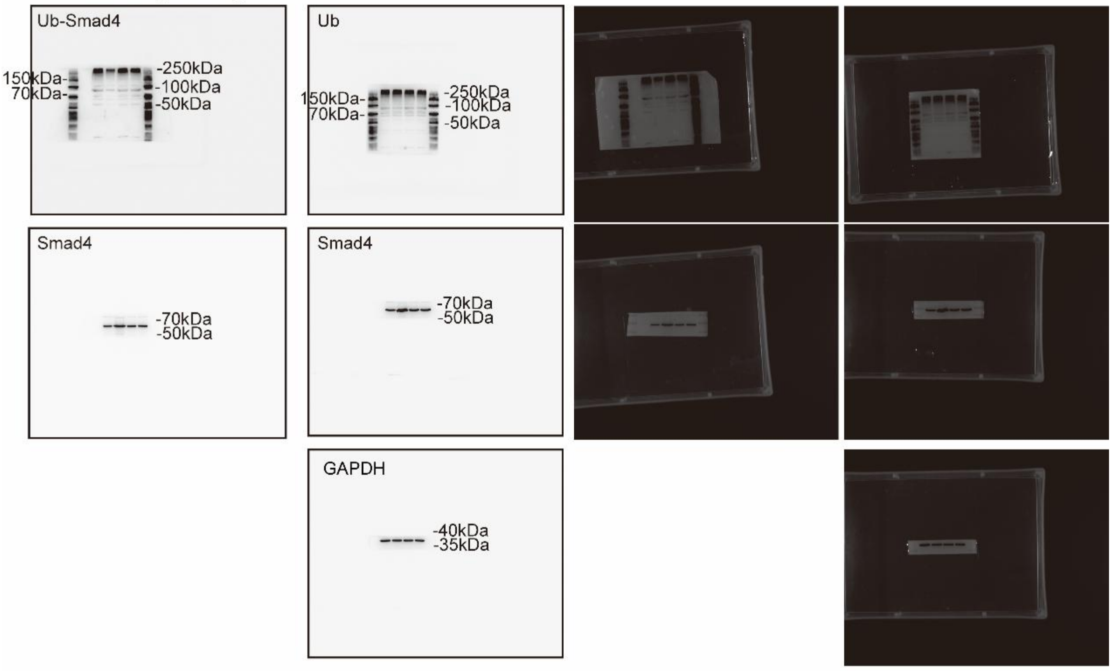

Full unedited gel for figure7H

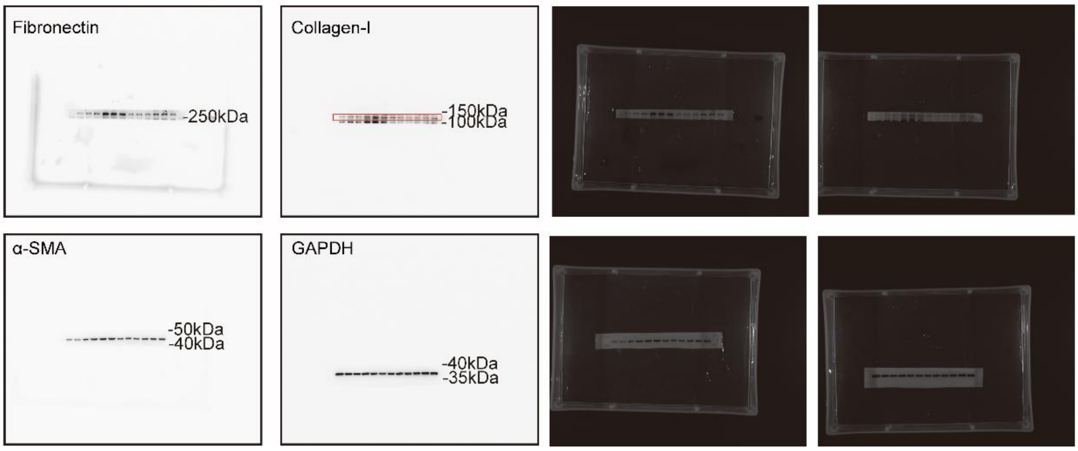

Full unedited gel for figure S4A

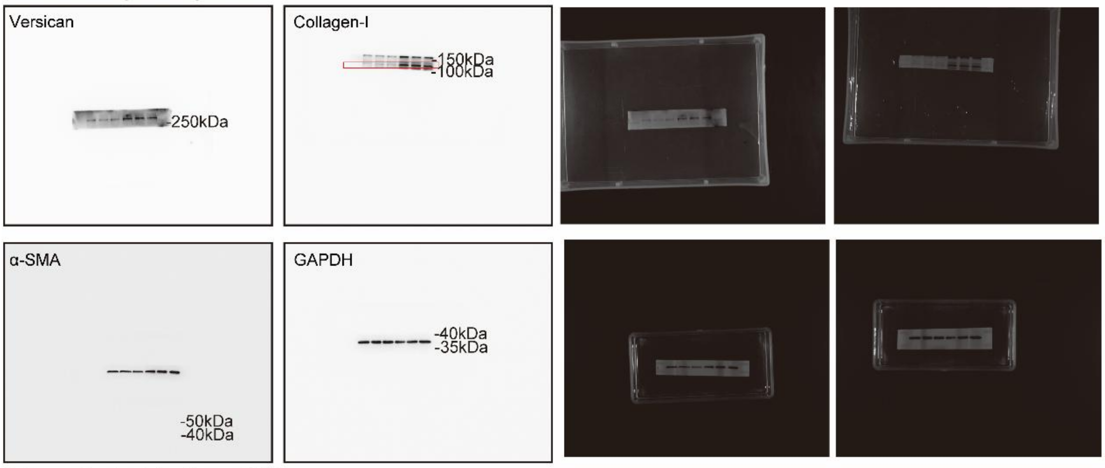

Full unedited gel for figure S4D

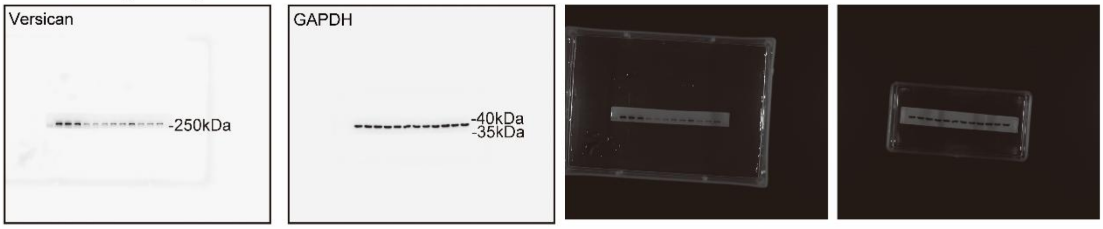

Full unedited gel for figure S4G

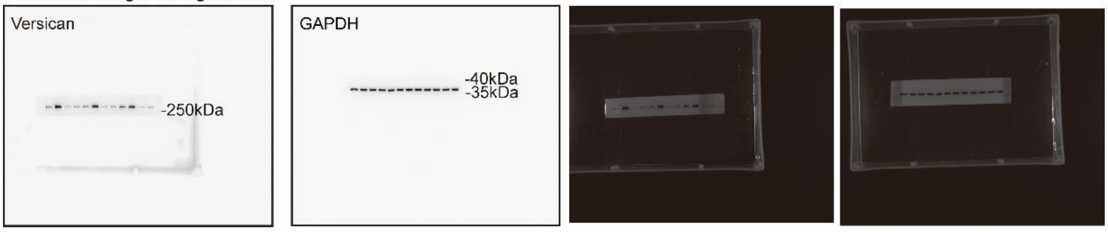

Full unedited gel for figure S4H

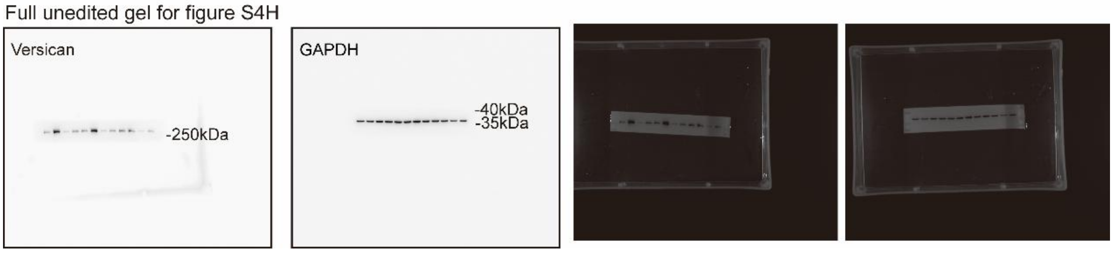

Full unedited gel for figure S5

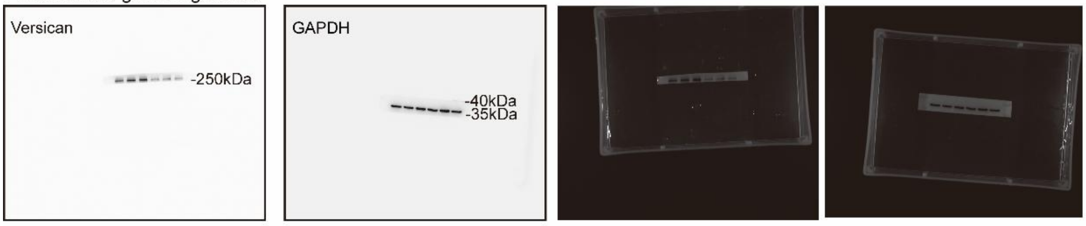

Full unedited gel for figure S6A

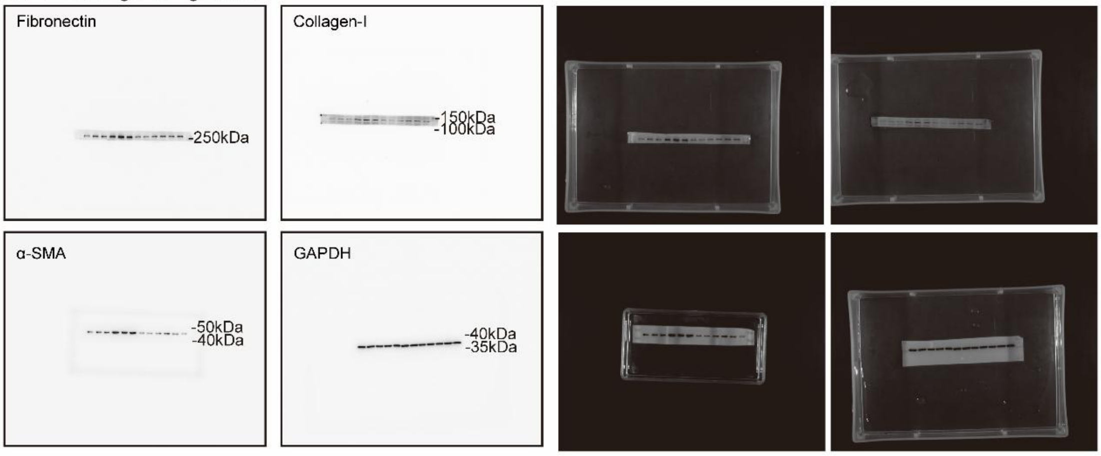

Full unedited gel for figure S6D

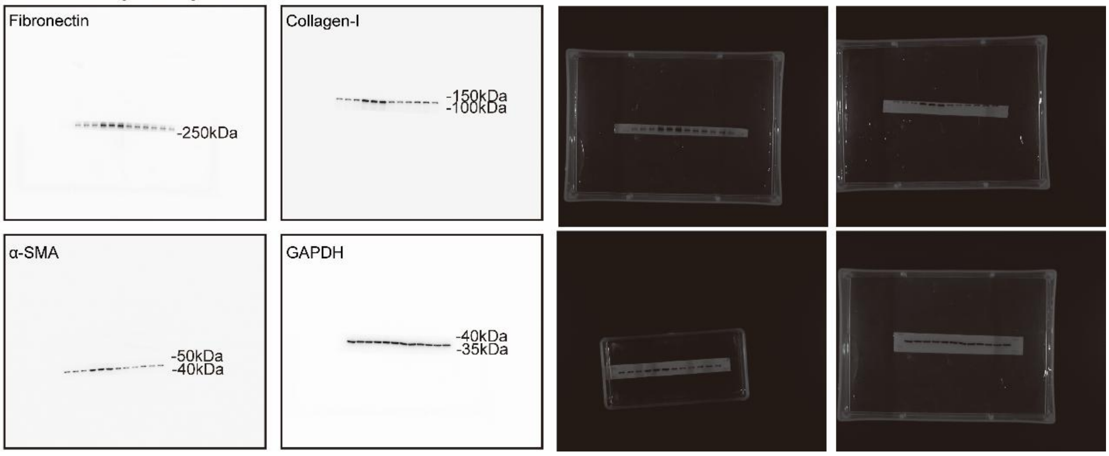

Full unedited gel for figure S6G

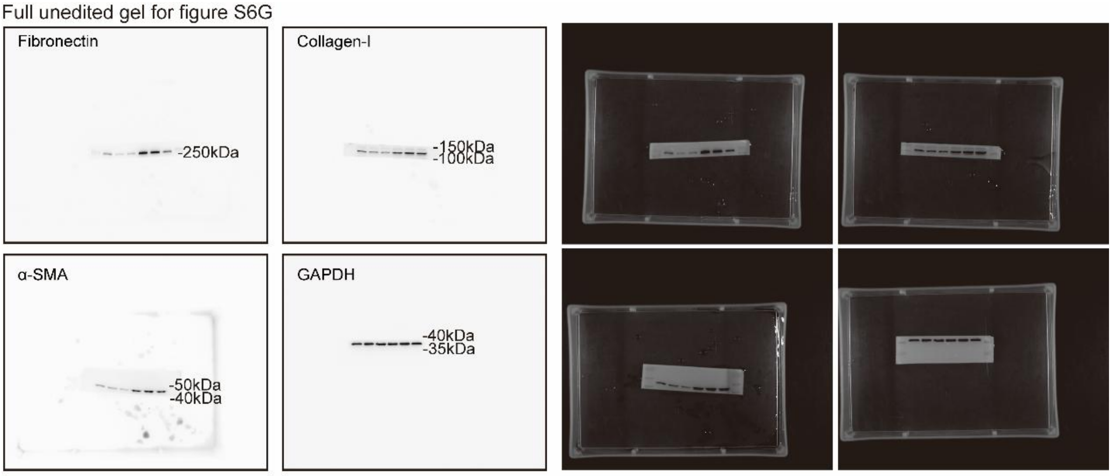

Full unedited gel for figure S7A

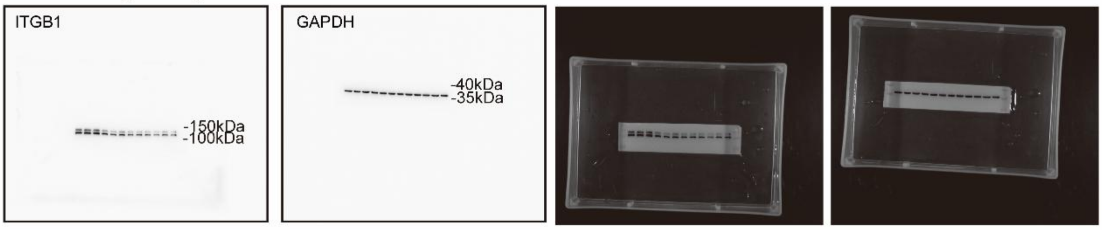

Full unedited gel for figure S7D

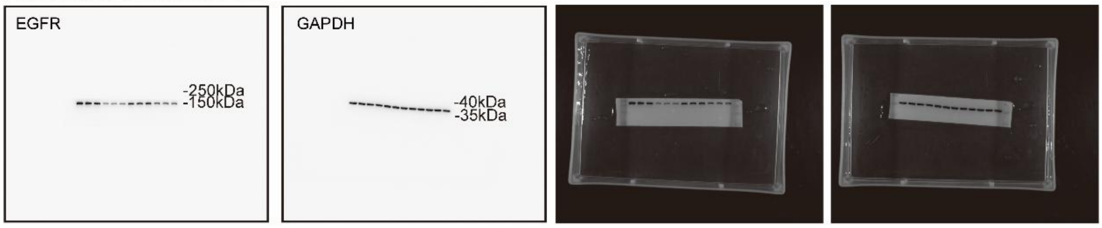

Full unedited gel for figure S7G

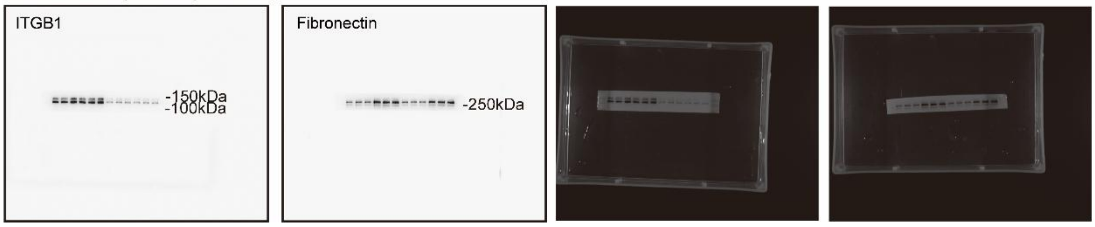

Full unedited gel for figure S7G

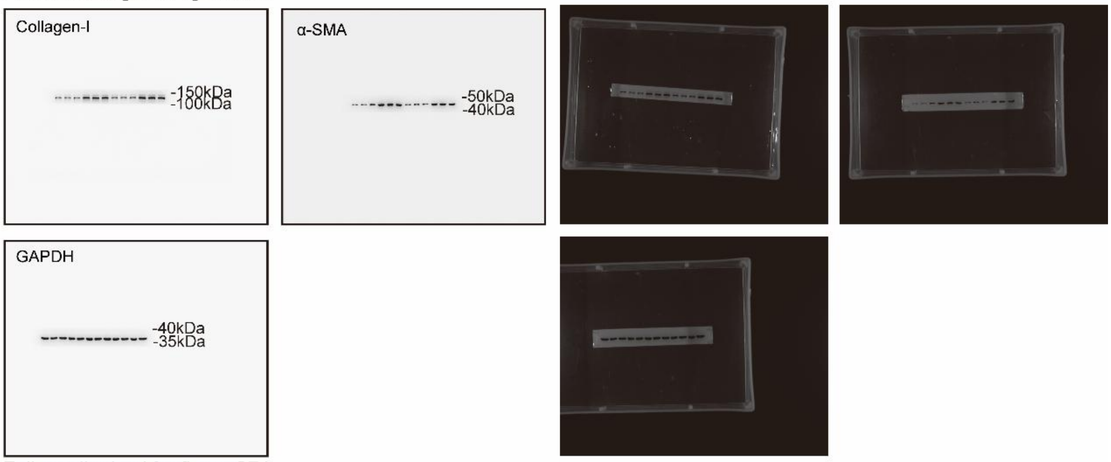

Full unedited gel for figure S7J

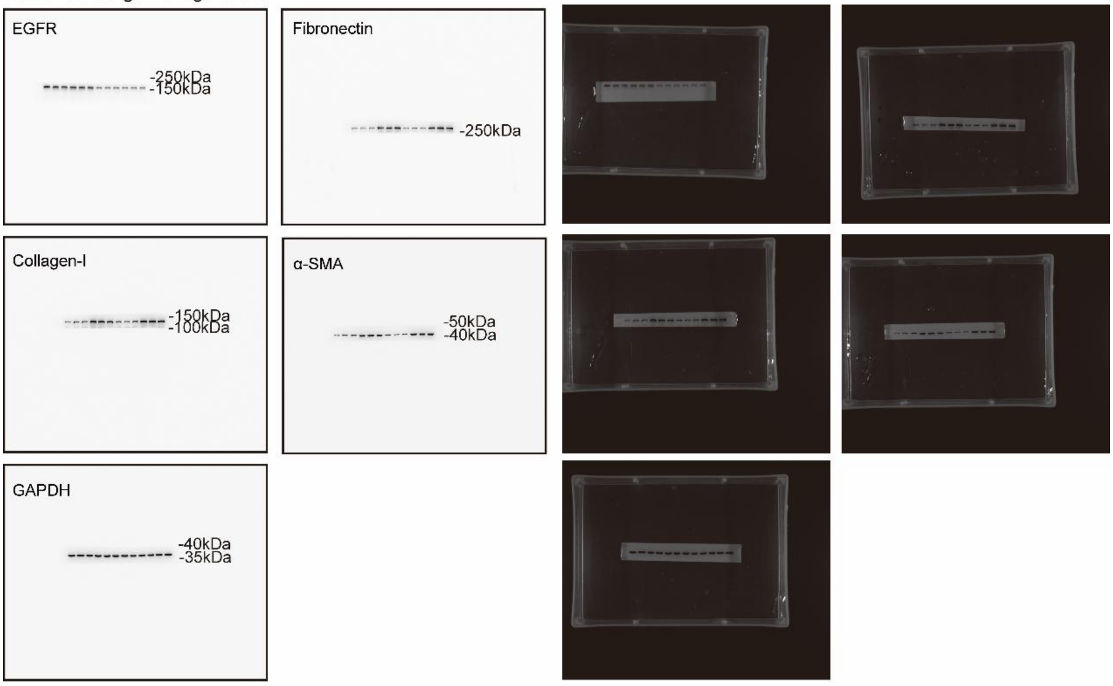

Full unedited gel for figure S8

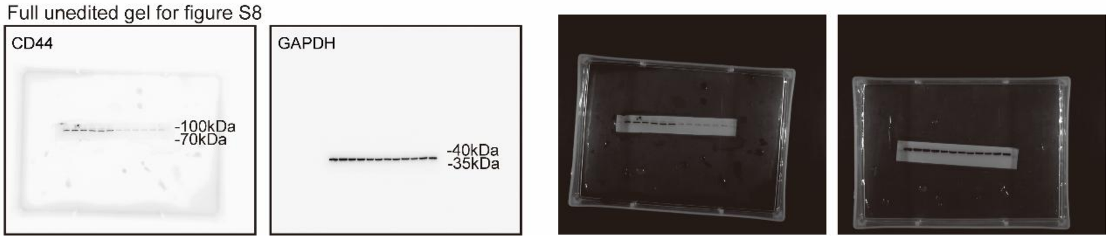

Full unedited gel for figure S11A

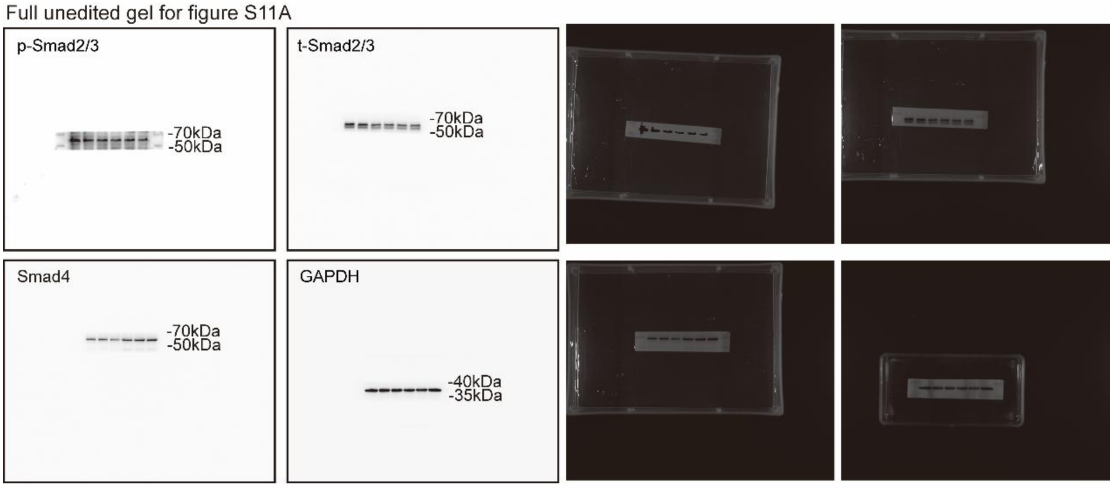

Full unedited gel for figure S11D

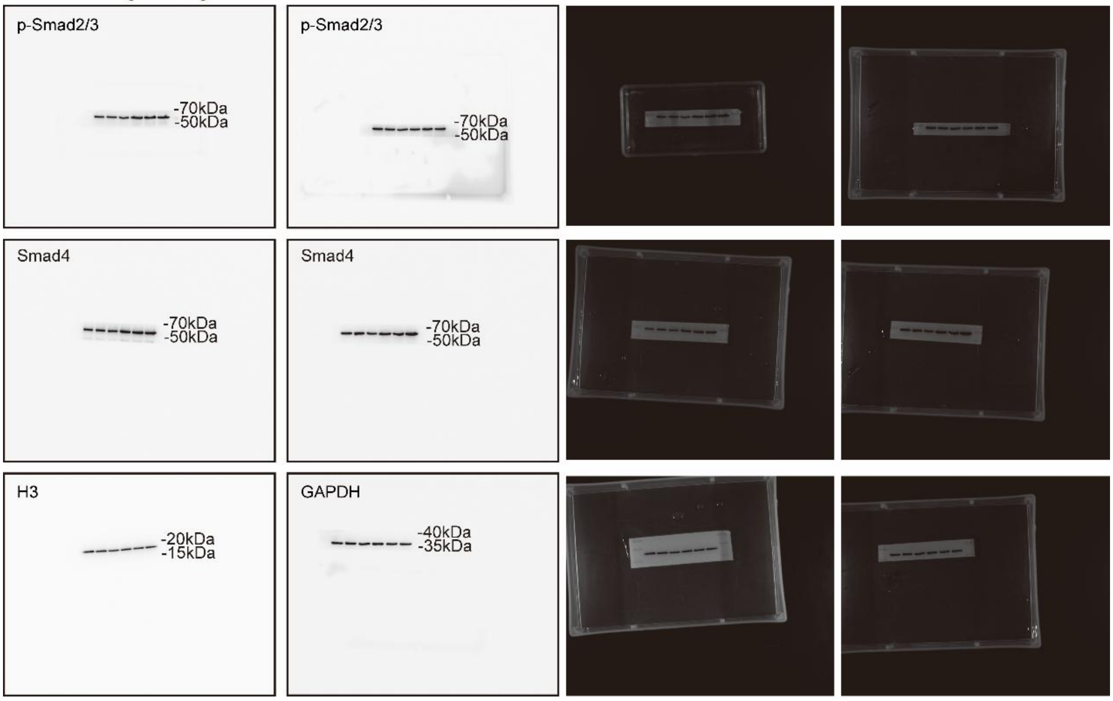

Full unedited gel for figure S12B

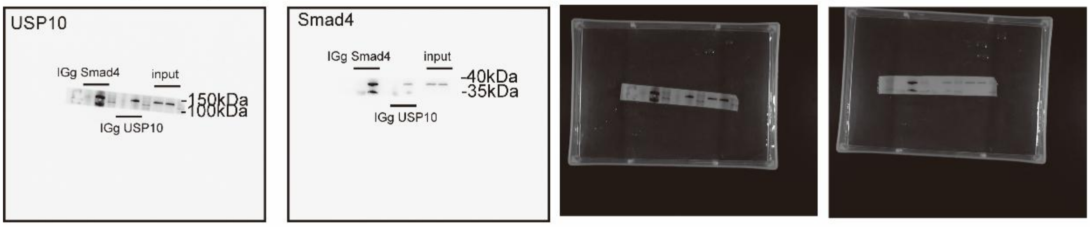

Full unedited gel for figure S13

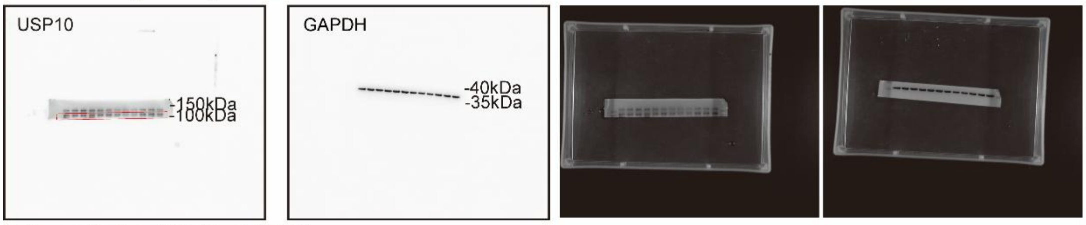

Full unedited gel for figure S15A

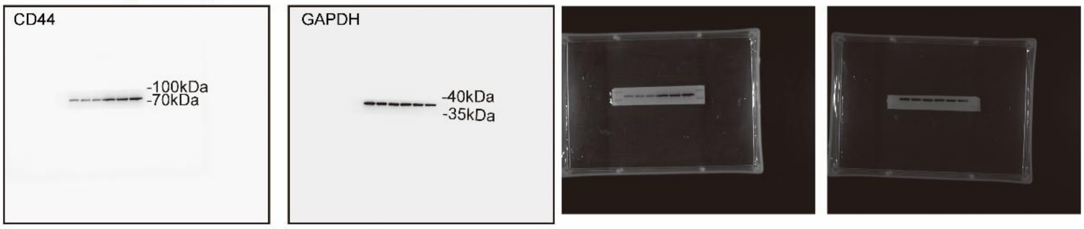

Full unedited gel for figure S15B

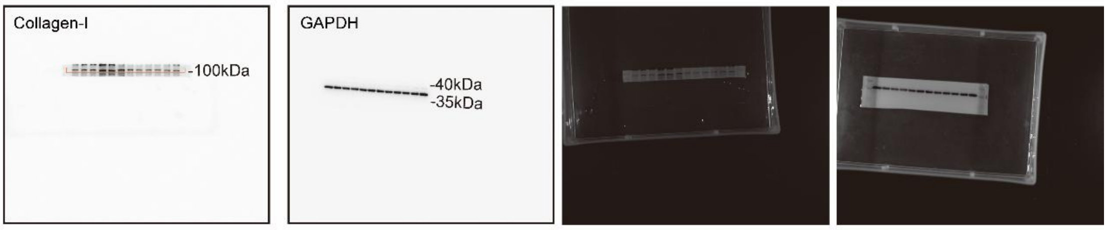

Full unedited gel for figure S15C

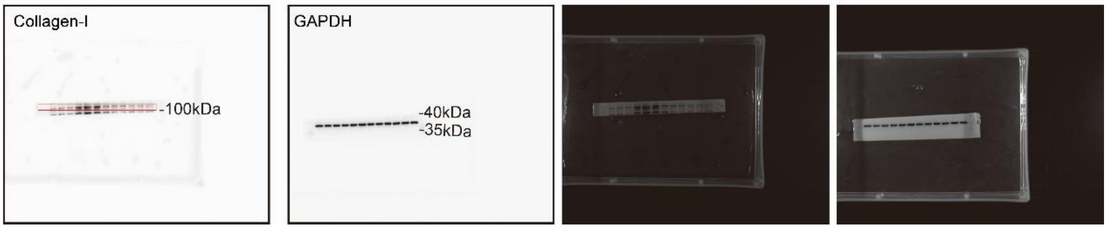

Full unedited gel for figure S16B

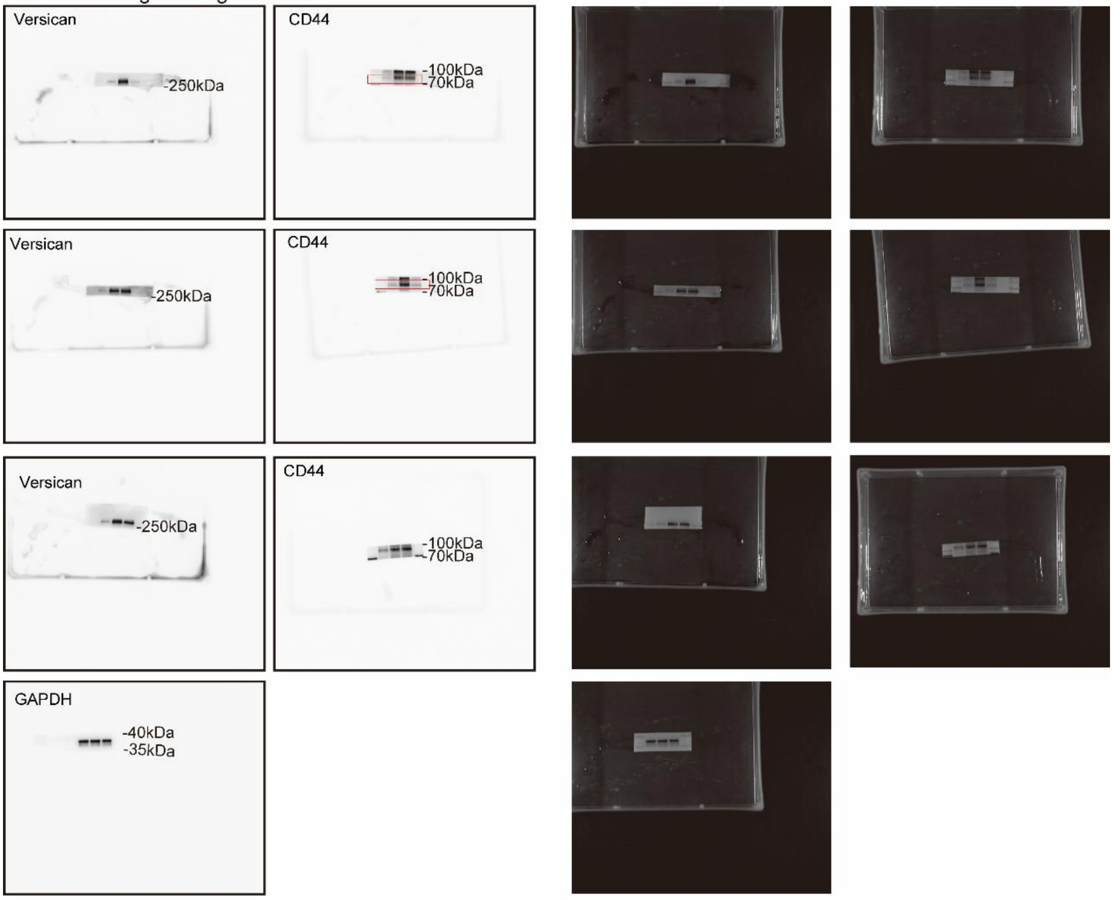

Full unedited gel for figure S16C

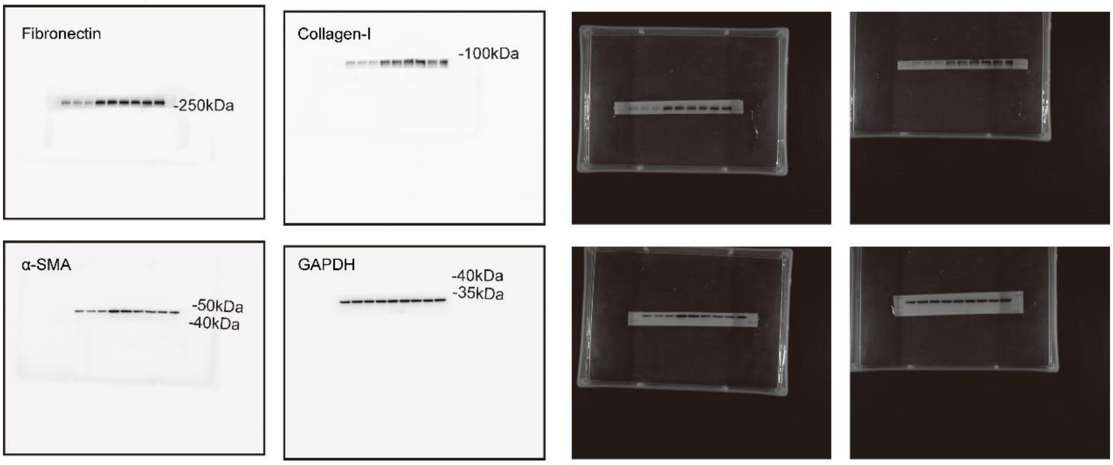

Full unedited gel for figure S16D

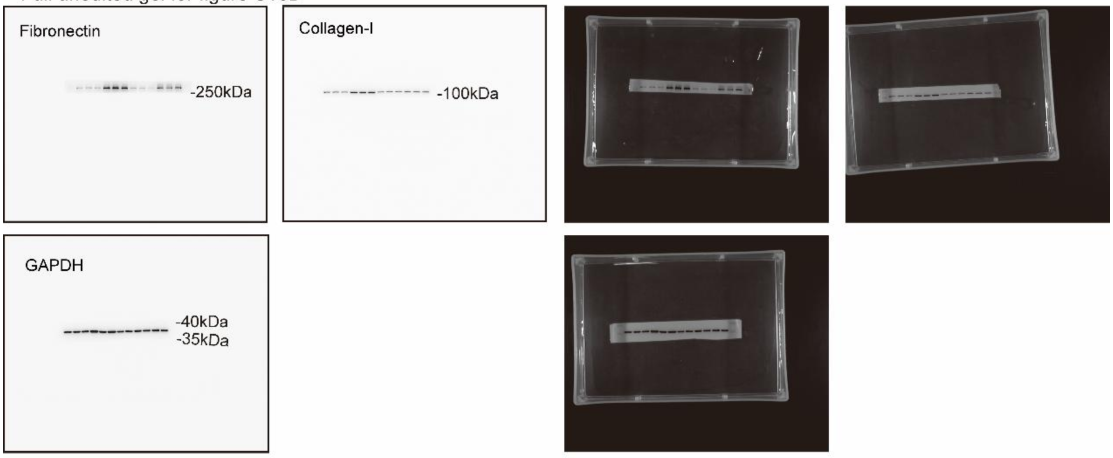

Full unedited gel for figure S16E

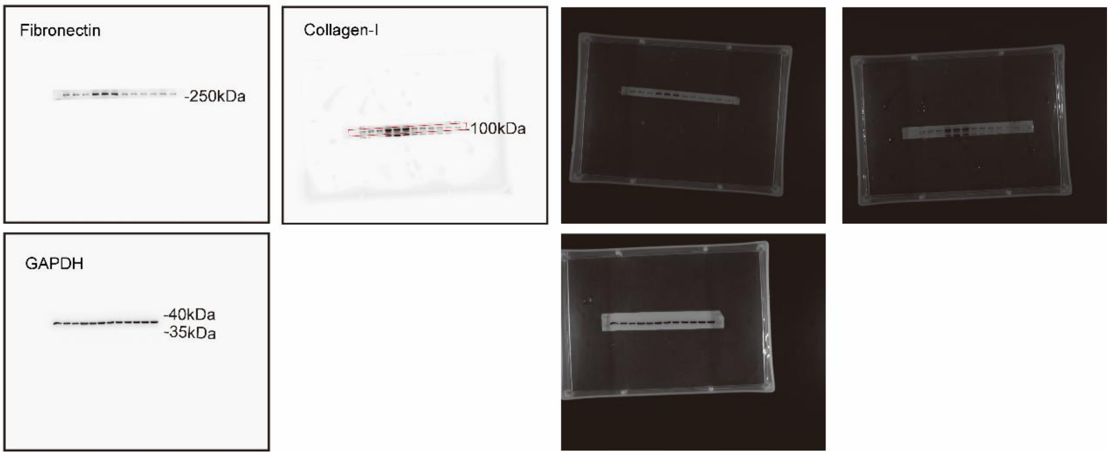

Supplement: Unedited blot and gel images [file jciinsight-11-199507-s083.pdf]
